# Demosaicing of CFA 3.0 with Applications to Low Lighting Images

**DOI:** 10.3390/s20123423

**Published:** 2020-06-17

**Authors:** Chiman Kwan, Jude Larkin, Bulent Ayhan

**Affiliations:** Applied Research LLC; Rockville, MD 20850, USA; judelarkin93@gmail.com (J.L.); bulent.ayhan@signalpro.net (B.A.)

**Keywords:** debayering, demosaicing, color filter array (CFA), RGBW pattern, Bayer pattern, CFA 1.0, CFA 2.0, CFA 3.0, pansharpening, deep learning

## Abstract

Low lighting images usually contain Poisson noise, which is pixel amplitude-dependent. More panchromatic or white pixels in a color filter array (CFA) are believed to help the demosaicing performance in dark environments. In this paper, we first introduce a CFA pattern known as CFA 3.0 that has 75% white pixels, 12.5% green pixels, and 6.25% of red and blue pixels. We then present algorithms to demosaic this CFA, and demonstrate its performance for normal and low lighting images. In addition, a comparative study was performed to evaluate the demosaicing performance of three CFAs, namely the Bayer pattern (CFA 1.0), the Kodak CFA 2.0, and the proposed CFA 3.0. Using a clean Kodak dataset with 12 images, we emulated low lighting conditions by introducing Poisson noise into the clean images. In our experiments, normal and low lighting images were used. For the low lighting conditions, images with signal-to-noise (SNR) of 10 dBs and 20 dBs were studied. We observed that the demosaicing performance in low lighting conditions was improved when there are more white pixels. Moreover, denoising can further enhance the demosaicing performance for all CFAs. The most important finding is that CFA 3.0 performs better than CFA 1.0, but is slightly inferior to CFA 2.0, in low lighting images.

## 1. Introduction

**Figure 1:**
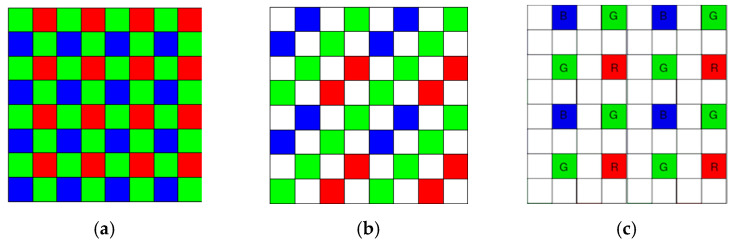
Three CFA patterns. (**a**) CFA 1.0; (**b**) CFA 2.0; (**c**) CFA 3.0.

Many commercial cameras have incorporated the Bayer pattern [1], which is also named as color filter array (CFA) 1.0. An example of CFA 1.0 is shown in Figure 1a. There are many repetitive 2 × 2 blocks and, in each block, two green, one red, and one blue pixels are present. To save cost, the Mastcam onboard the Mars rover Curiosity [2,3,4,5] also adopted the Bayer pattern. Due to the popularity of CFA 1.0, Kodak researchers invented a red-green-blue-white (RGBW) pattern or CFA 2.0 [6,7]. An example of the RGBW pattern is shown in Figure 1b. In each 4 × 4 block, eight white pixels, four green pixels, and two red and blue pixels are present. Numerous other CFA patterns have been invented in the past few decades [8,9,10]. 

Researchers working on CFAs believe that CFA 2.0 is more suitable for taking images in low lighting environments. Recently, some researchers [11] have further explored the possibility of adding more white pixels to the CFA 2.0. The new pattern has 75% white pixels and the RGB pixels are randomly distributed among the remaining 25% pixels. 

Motivated by the work in [11], we propose a simple CFA pattern in which the RGB pixels are evenly distributed, instead of using randomly distributed RGB pixels. In particular, as shown in Figure 1c, each 4 × 4 block has 75% or 12 white pixels, 12.5% or two green pixels, 6.25% or one red and blue pixels. We identify this CFA pattern as the CFA 3.0. There are three key advantages of using fixed CFA patterns. For the random pattern case, each camera will have a different pattern. In contrast, the first advantage is that the proposed fixed pattern allows a camera manufacturer to mass produce the cameras without changing the RGBW patterns for each camera. This can save manufacturing cost quite significantly. The second advantage is that the demosaicing software can be the same in all cameras if the pattern is fixed. Otherwise, each camera needs to have a unique demosaicing software tailored to a specific random pattern. This will seriously affect the cost. The third advantage is that some of the demosaicing algorithms for CFA 2.0 can be applied with little modifications. This can be easily seen if one puts the standard demosaicing block diagrams for CFA 2.0 and CFA 3.0 side by side. One can immediately notice that the reduced resolution color image and the panchromatic images can be similarly generated. As a result, the standard approach for CFA 2.0, all the pan-sharpening based algorithms for CFA 2.0, and the combination of pan-sharpening, and deep learning approaches for CFA 2.0, that we developed earlier in [12] can be applied to CFA 3.0. 

In our recent paper on the demosaicing of CFA 2.0 (RGBW) [12], we have compared CFA 1.0 and CFA 2.0 using IMAX and Kodak images and observed that CFA 1.0 was better than CFA 2.0. One may argue that our comparison was not fair because IMAX and Kodak datasets were not collected in low lighting conditions and CFA 2.0 was designed for taking images in low lighting environments. Due to the dominance of white pixels in CFA 2.0, the SNR of the collected image is high and hence CFA 2.0 should have better demosaicing performance in dark environments.

Recently, we systematically and thoroughly compared CFA 1.0 and CFA 2.0 under dark conditions [13]. We observed that CFA 2.0 indeed performed better under dark conditions. We also noticed that denoising can further improve the demosaicing performance. 

The aforementioned discussions immediately lead to several questions concerning the different CFAs. First, we enquired how demosaic CFA 3.0. Although there are universal debayering algorithms [8,9,10], those codes are not accessible to the public or may require customization. Here, we propose quite a few algorithms that can demosaic CFA 3.0, and this can be considered as our first contribution. Second, regardless of whether the answer to the first question is positive or negative, will more white pixels in the CFA pattern help the demosaicing performance for low lighting images? In other words, will CFA 3.0 have any advantages over CFA 1.0 and CFA 2.0? It will be a good contribution to the research community to answer the question: Which CFA out of the three is most suitable for low lighting environments? Third, the low lighting images contain Poisson noise and demosaicing does not have denoising capability. To improve the demosaicing performance, researchers usually carry out some denoising and contrast enhancement. It is important to know where one should perform denoising. Denoising can be performed either after or before demosaicing. Which choice can yield better overall image quality? Answering the above questions will assist designers understand the next generation of cameras that have adaptive denoising capability to handle diverse lighting environments.

In this paper, we will address the aforementioned questions. After some extensive research and experiments, we found that some algorithms for CFA 2.0 can be adapted to demosaic CFA 3.0. For instance, the standard approach for CFA 2.0 is still applicable to CFA 3.0. The pan-sharpening based algorithms for CFA 2.0 [12] and deep learning based algorithms for CFA 2.0 [14] are also applicable to CFA 3.0. We will describe those details in Section 2. In Section 3, we will first present experiments to demonstrate that CFA 3.0 can work well for low lighting images. Denoising using block matching in 3D (BM3D) [15] can further enhance the demosaicing performance. We also summarize a comparative study that compares the performance of CFA 1.0, CFA 2.0, and CFA 3.0 using normal and emulated low lighting images. We have several important findings. First, having more white pixels does not always improve the demosaicing performance. CFA 2.0 achieved the best performance. CFA 3.0 performs better than CFA 1.0 and is slightly inferior to CFA 2.0. Second, denoising can further enhance the demosaicing performance in all CFAs. Third, we observed that the final image quality relies heavily on the location of denoising. In particular, denoising after demosaicing is worse than denoising before demosaicing. Fourth, when the SNR is low, denoising has more influence on demosaicing. Some discussions on those findings are also included. In Section 4, some remarks and future research directions will conclude our paper.

## 2. Demosaicing Algorithms

We will first review some demosaicing algorithms for CFA 2.0. We will then answer the first question mentioned in Section 1: how one can demosaic the CFA 3.0 pattern shown in Figure 1c. It turns out that some of the existing algorithms for CFA 2.0 can be used for CFA 3.0 with some minor modifications.

### 2.1. Demosaicing Algorithms for CFA 2.0

The baseline approach is a simple demosaicing operation on the CFA, followed by an upsampling of the reduced resolution color image shown in Figure 2 of [13]. The standard approach consists of four steps as shown in Figure 2 of [13]. Step 1 interpolates the luminance image with half of the white pixels missing. Step 2 subtracts the reduced color image from the down-sampled interpolated luminance image. Step 3 upsamples the difference image in Step 2. Step 4 fuses the full resolution luminance with the upsampled difference image in Step 3. In our implementation, the demosaicing of the reduced resolution color image is done using local directional interpolation and nonlocal adaptive thresholding (LDI-NAT) [16] and the pan interpolation is also done using LDI-NAT [16].

In our recent paper [12], a pan-sharpening approach, as shown in Figure 2 of [13], was proposed to demosaicing CFA 2.0. The demosaicing of the reduced resolution color image is done using LDI-NAT [16]. The panchromatic (luminance) band with missing pixels is interpolated using LDI-NAT [16]. After those steps, pan-sharpening is performed to generate the full resolution color image. It should be noted that many pan-sharpening algorithms have been used in our experiments, including Principal Component Analysis (PCA) [17], Smoothing Filter-based Intensity Modulation (SFIM) [18], Modulation Transfer Function Generalized Laplacian Pyramid (GLP) [19], MTF-GLP with High Pass Modulation (HPM) [20], Gram Schmidt (GS) [21], GS Adaptive (GSA) [22], Guided Filter PCA (GFPCA) [23], PRACS [24] and hybrid color mapping (HCM) [25,26,27,28,29].

In a recent paper by us [14], the pan-sharpening approach has been improved by integrating with deep learning. As shown in Figure 4 of [13], a deep learning method was incorporated in two places. First, deep learning has been used to demosaic the reduced resolution CFA image. Second, deep learning has been used to improve the interpolation of the pan band. We adopted a deep learning algorithm known as Demonet [30]. Good performance improvement has been observed.

Moreover, the least-squares luma-chroma demultiplexing (LSLCD) [31] algorithm was used in our experiments for CFA 2.0.

In the past, we also developed two pixel-level fusion algorithms known as fusion of 3 (F3) algorithms and alpha trimmed mean filter (ATMF), which were used in our earlier studies [12,13,14,32]. Three best performing algorithms are fused in F3 and seven high performing algorithms are fused in ATMF. These fusion algorithms are applicable to any CFAs.

### 2.2. Demosaicing Algorithms for CFA 3.0

**Figure 2:**
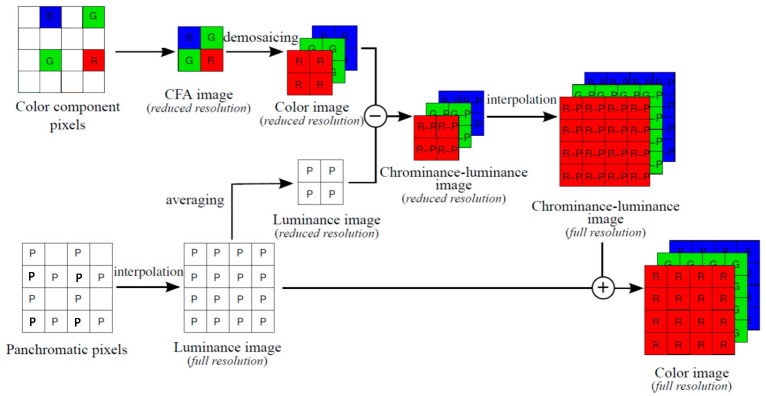
Standard approach for CFA 3.0.

As opposed to the random color patterns in [11], the CFA 3.0 pattern in this paper has fixed patterns. One key advantage is that some of the approaches for CFA 2.0 can be easily applied with little modifications. For instance, the standard approach shown in Figure 2 of [13] for CFA 2.0 can be immediately applied to CFA 3.0, as shown in Figure 2. In each 4 × 4 block, the four R, G, B pixels in the CFA 3.0 raw image are extracted to form a reduced resolution CFA image. A standard demosaicing algorithm, any of those mentioned in Section 2.1 can be applied. In our implementation, we used LDI-NAT [16] for demosaicing the reduced resolution color image. The missing pan pixels are interpolated using LDI-NAT [16] to create a full resolution pan image. The subsequent steps will be the same as before.

**Figure 3:**
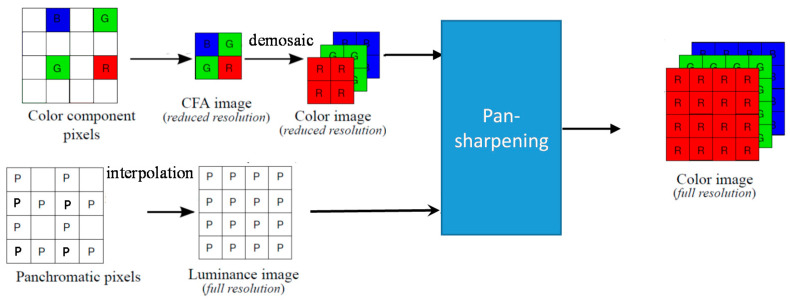
A pan-sharpening approach for CFA 3.0.

Similarly, the pan-sharpening approach for CFA 2.0 shown in Figure 3 of [13] can be applied to CFA 3.0 as shown in Figure 3. Here, the four R, G, B pixels are extracted first and then a demosaicing algorithm for CFA 1.0 is applied to the reduced resolution Bayer image. We used LDI-NAT [16] for reduced resolution color image. For the pan band, any interpolation algorithms can be applied. We used LDI-NAT. Afterwards, any pan-sharpening algorithms mentioned earlier can be used to fuse the pan and the demosaiced reduced resolution color image to generate a full resolution color image. In our experiments, we have used PCA [17], SFIM [18], GLP [19], HPM [20], GS [21], GSA [22], GFPCA [23], PRACS [24] and HCM [25] for pan-sharpening.

**Figure 4:**
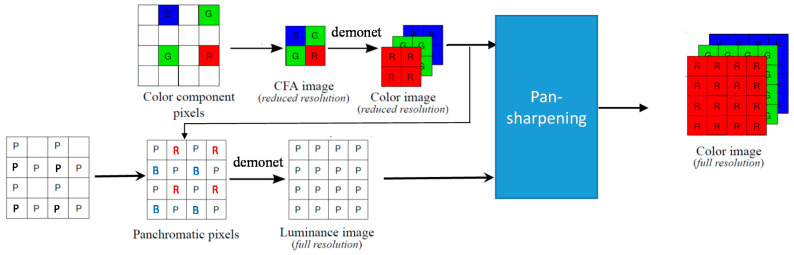
A hybrid deep learning and pan-sharpening approach for CFA 3.0.

The hybrid deep learning and pan-sharpening approach for CFA 2.0 shown in Figure 4 of [13] can be extended to CFA 3.0, as shown in Figure 4. For the reduced resolution demosaicing step, the Demonet algorithm is used. In the pan band generation step, we also propose to apply Demonet. The details are similar to our earlier paper on CFA 2.0 [12]. Hence, we skip the details. After those two steps, a pan-sharpening algorithm is then applied. In our experiments, Demonet is combined with different pan-sharpening algorithms in different scenarios. For normal lighting conditions, GSA is used for pan-sharpening and we call this hybrid approach the Demonet + GSA method. For low lighting conditions, it is more effective to use GFPCA for pan-sharpening and we term this as the Demonet + GFPCA method.

The two fusion algorithms (F3 and ATMF) can be directly applied to CFA 3.0.

### 2.3. Performance Metrics

Five performance metrics were used in our experiments to compare the different methods and CFAs. These metrics are well-known in the literature.

- Peak Signal-to-Noise Ratio (PSNR) [33]
  Separate PSNRs in dBs are computed for each band. A combined PSNR is the average of the PSNRs of the individual bands. Higher PSNR values imply higher image quality.
- Structural SIMilarity (SSIM)
  In [34], SSIM was defined to measure the closeness between two images. An SSIM value of 1 means that the two images are the same.
- Human Visual System (HVS) metric
  Details of HVS metric in dB can be found in [35].
- HVSm (HVS with masking) [36]
  Similar to HVS, HVS incorporates the visual masking effects in computing the metrics.
- CIELAB
  We also used CIELAB [37] for assessing demosaicing and denoising performance in our experiments.

## 3. Experiments

In Section 2, we answer the first question about how one can demosaic CFA 3.0. Here, we will answer the two remaining questions mentioned in Section 1. One of the questions is whether or not the new CFA 3.0 can perform well for demosacing low lighting images. The other question is regarding whether CFA 3.0 has any advantages over the other two CFAs. Simply put, we will answer which one of the three CFAs is the best method for low light environments.

### 3.1. Data

A benchmark dataset (Kodak) was downloaded from a website (http://r0k.us/graphics/kodak/) and 12 images were selected. The images are shown in Figure 5 of [13]. We will use them as reference images for generating objective performance metrics. In addition, noisy images emulating images collected from dark conditions will be created using those clean images. It should be noted that the Kodak images were collected using films and then converted to digital images. We are absolutely certain that the images were not created using CFA 1.0. Many researchers in the demosaicing community have used Kodak data sets in their studies.

Emulating images in low lighting conditions is important because ground truth (clean) images can then be used for a performance assessment. In the literature, some researchers used Gaussian noise to emulate low lighting images. We think the proper way to emulate low lighting images is by using Poisson noise, which is simply because the noise introduced in low lighting images follows a Poisson distribution.

The differences between Gaussian and Poisson noises are explained as follows. Gaussian noise is additive, independent at each pixel, and independent of the pixel intensity. It is caused primarily by Johnson–Nyquist noise (thermal noise) [38]. Poisson noise is pixel intensity dependent and is caused by the statistical variation in the number of photons. Poisson noise is also known as photon shot noise [39]. As the number of photons at the detectors of cameras follows a Poisson distribution, and hence, the name of Poisson noise, when the number of photons increases significantly, the noise behavior then follows a Gaussian distribution due to the law of large numbers. However, the shot noise behavior of transitioning from Poisson distribution to Gaussian distribution does not mean that Poisson noise (photon noise) becomes Gaussian noise (thermal noise) when the number of photons increases significantly. This may be confusing for many people due to the terminology of Gaussian distribution. In short, the two noises come from different origins and have very different behaviors.

Poisson distribution has been widely used to characterize discrete events. For example, the arrival of customers to a bank follows a Poisson distribution; the number of phone calls to a cell phone tower also follows a Poisson distribution. For cameras, the probability density function (pdf) of photon noise in an image pixel follows a Poisson distribution, which can be mathematically described as,

(1) $$P\left(k\right)=\frac{\lambda^{k}e^{-\lambda }}{k!}$$

where *λ* is the mean number of photons per pixel and *P*(*k*) is the probability when there are *k* photons. Based on the above pdf, one can interpret the actual number of photons arriving at a detector pixel fluctuates around the mean (*λ*), which can be used to characterize the lighting conditions. That is, a small *λ* implies the lighting is low and vice versa.

**Figure 5:**
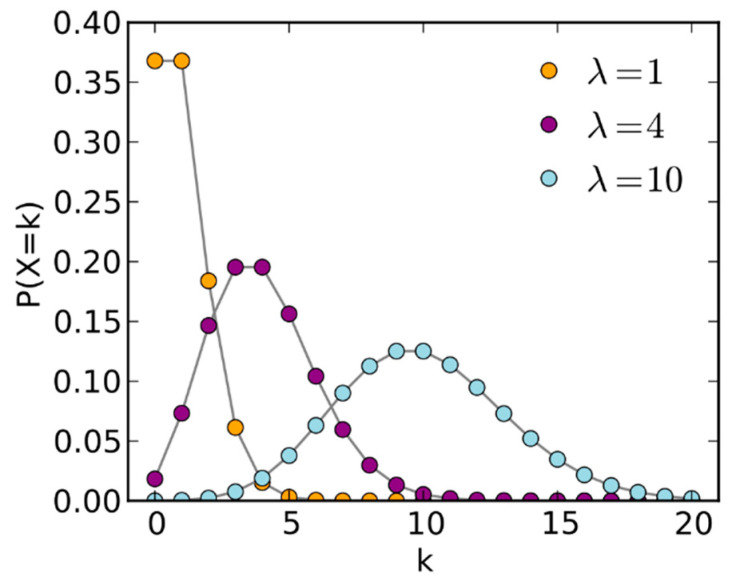
Poisson distributions with varying λ.

In statistics, when *λ* increases to a large number, the pdf in (1) will become a continuous pdf known as the Gaussian distribution, which is given by,

(2) $$P\left(x\right)=\frac{1}{\sqrt{2\pi \lambda }}e^{-\frac{1}{2\lambda }{\left(x-\lambda \right)}^{2}}$$

where *x* denotes the continuous noise variable and *λ* is the same for both mean and variance in Poisson noise. In [40], central limit theorem is used to connect (1) and (2) by assuming *λ* >> 1. The derivation of (2) from (1) can be found in [41]. Figure 5 [42] clearly shows that the Poisson distribution gradually becomes a Gaussian distribution when *λ* increases. It appears that when *λ* = 10, the Poisson pdf already looks like a Gaussian distribution.

However, it must be emphasized here that although (2) follows the normal or Gaussian distribution, the noise is still photon shot noise, not Gaussian noise due to thermal noise.

In contrast, Gaussian noise (thermal noise) follows the following distribution,

(3) $$P\left(z\right)=\frac{1}{\sqrt{2\pi }\sigma }e^{-\frac{1}{2}{\left(\frac{z-\mu }{\sigma }\right)}^{2}}$$

where *z* is the noise variable, *μ* is the mean, and *σ* is the standard deviation. As mentioned earlier, Gaussian noise is thermal noise and is independent of pixels and pixel intensity. To introduce Gaussian noise to a clean image, one can use a Matlab function: imnoise (*I*, “Gaussian”, *μ*, *σ*^2^) where *I* is a clean image and *μ* is set to zero.

Here, we describe a little more about the imaging model in low lighting conditions. As mentioned earlier, Poisson noise is related to the average number of photons per pixel, *λ*. To emulate the low lighting images, we vary *λ*. It should be noted that the SNR in dB of a Poisson image is given by [40]:

(4) SNR=10log(λ)

A number of images with different levels of Poisson noise or SNR can be seen in the table in Appendix A.

The process of how we introduced Poisson noise is adapted from code written by Erez Posner (https://github.com/erezposner/Shot-Noise-Generator) and it is summarized as follows.

Given a clean image and the goal of generating a Poisson noisy image with a target signal-to-noise (SNR) value in dB, we first compute the full-well (FW) capacity of the camera, which is related to the SNR through:

(5) $$\mathrm{FW}=10^{\mathrm{SNR}/10}$$

For a pixel *I*(*i*,*j*), we then compute the average photons per pixel (*λ*) for that pixel by,

*λ* = *I*(*i*,*j*) × *FW*/255 (6)

where 255 is the number of intensity levels in an image. Using a Poisson noise function created by Donald Knuth [43], we can generate an actual photon number *k* through the Poisson distribution described by Equation (1). This *k* value changes randomly whenever a new call to the Poisson noise function is being made.

Finally, the actual noisy pixel amplitude (*I_n_*(*i*,*j*)) is given by:

*I_n_*(*i*,*j*) = 255 × *k*/*FW* (7)

A loop iterating over every (*i*,*j*) in the image will generate the noisy Poisson image with the target SNR value.

Although Gaussian and Poisson noises have completely different characteristics, it will be interesting to understand when the two noises will become indistinguishable. To achieve that and to save some space, we include noisy images between 20 dBs and 38 dBs. It should be noted that the Gaussian noise was generated using Matlab’s noise generation function (imnoise). The Poisson noise was generated following an open source code [44]. The SNRs are calculated by comparing the noisy images to the ground truth image. From Table A1 in Appendix A, we can see that when SNR values are less than 35 dBs, the two types of noisy images are visually different. Poisson images are slightly darker than Gaussian images. When SNR increases beyond 35 dBs, the two noisy images are almost indistinguishable.

From this study, we can conclude that 35-dB SNR is the threshold for differentiating Poisson noise (photon shot noise) from Gaussian noise (thermal). At 35 dBs, the average number of photons per pixel arriving at the detector is 3200 for Poisson noise and the standard deviation of the Gaussian noise is 0.0177. The image pixels are in double precision and normalized between 0 and 1.

**Figure 6:**
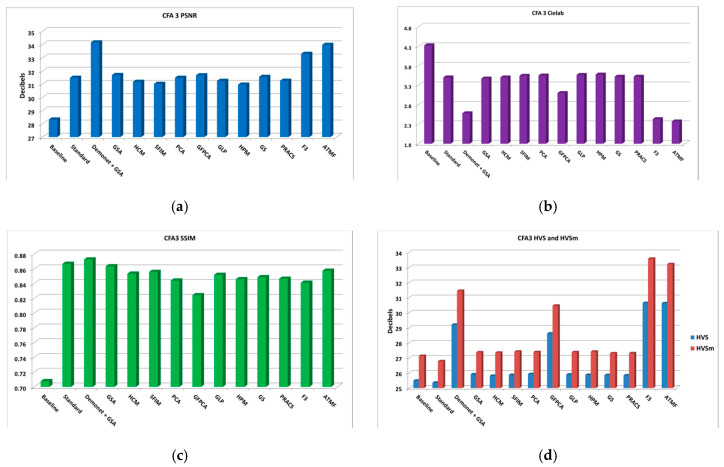
Averaged performance metrics for all the clean images. (**a**) PNSR; (**b**) CIELAB; (**c**) SSIM; (**d**) HVS and HVSm.

To create a consistent level of noise close to our SNR levels of 10 dBs and 20 dBs, we followed a technique described in [44,45]. For each color band, we added Poisson noise separately. The noisy and low lighting images at 10 dBs and 20 dBs are shown in Figures 6 and 7 of [13], respectively.

In this paper, denoising is done via BM3D [15], which is a well-known method in the research community. The particular BM3D is specifically for Poisson noise. We performed denoising in a band by band manner. The BM3D package we used is titled ‘Denoising software for Poisson and Poisson-Gaussian data,” released on March 16th 2016. See the link (http://www.cs.tut.fi/~foi/invansc/). We used this code as packaged, which requires the input to be a single band image. This package would not require any input other than a single band noisy image. We considered using the standard BM3D package titled “BM3D Matlab” in this link (http://www.cs.tut.fi/~foi/GCF-BM3D/) released on February 16th 2020. This package would allow denoising 3-band RGB images. This package, however, assumes Gaussian noise and required a parameter based on the noise level.

### 3.2. CFA 3.0 Results

Here, we will first present demosaicing of CFA 3.0 for clean images, which are collected under normal lighting conditions. We will then present demosaicing of low lighting images at two SNRs with and without denoising.

#### 3.2.1. Demosaicing Clean Images

There are 14 methods in our study. The baseline and standard methods are mentioned in Section 2.2. The other 12 methods include two fusion methods, one deep learning (Demonet + GSA), and nine pansharpening methods.

The three best methods used for F3 are Demonet + GSA, GSA, and GFPCA. The ATMF uses those three methods as well as Standard, PCA, GS, and PRACS.

From the PSNR and SSIM metrics in Table A2, the best performing algorithm is the Demonet + GSA method. The fusion methods of F3 and ATMF have better scores in Cielab, HVS and HVSm. Figure 6 shows the averaged metrics for all images.

**Figure 7:**
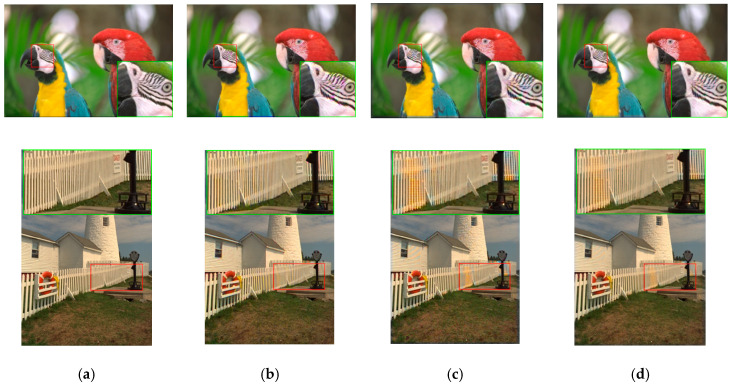
Visual comparison of three high performing demosaicing algorithms. The top row is the bird image and the bottom row is the lighthouse image. (**a**) Ground Truth; (**b**) Demonet + GSA; (**c**) ATMF; (**d**) F3.

In subjective comparisons shown in Figure 7, we can see the performance of the three selected methods (Demonet + GSA, ATMF and F3) varies a lot. Visually speaking, Demonet + GSA has the best visual performance. There are some minor color distortions in the fence area of the lighthouse image for F3 and ATMF.

#### 3.2.2. 10 dBs SNR

There are three cases in this sub-section. In the first case, we focus on the noisy images and there is no denoising. The second case includes denoising after demosaicing operation. The third case is about denoising before demosaicing operation.

● Case 1: No Denoising

There are 14 methods for demosaicing CFA 3.0. The F3 method is a fusion method that fused the results of Standard, Demonet+GFPCA, and GFPCA, which are the best performing individual methods for this case. The ATMF fusion method used the seven high performing methods, which are Standard, Demonet+GFPCA, GFPCA, Baseline, PCA, GS, and PRACS. Table A3 in Appendix A summarizes the PSNR, the CIELAB, SSIM, HVS, and HVSm metrics. The PSNR and CIELAB values vary a lot. All the SSIM, HVS, and HVSm values are not high.

**Figure 8:**
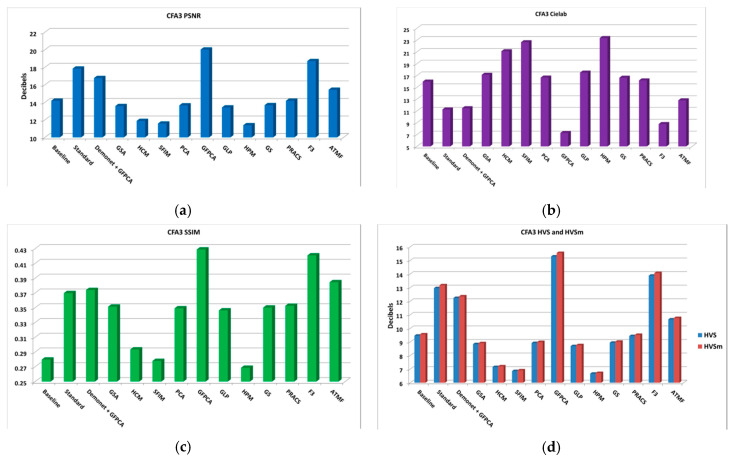
Averaged performance metrics for all the low lighting images at 10 dBs SNR (Poisson noise). (**a**) PNSR; (**b**) CIELAB; (**c**) SSIM; (**d**) HVS and HVSm.

The averaged PSNR, CIELAB, SSIM, HVS, and HVSm scores of all the 14 methods are shown in Figure 8. Big variations can be observed in the metrics.

**Figure 9:**
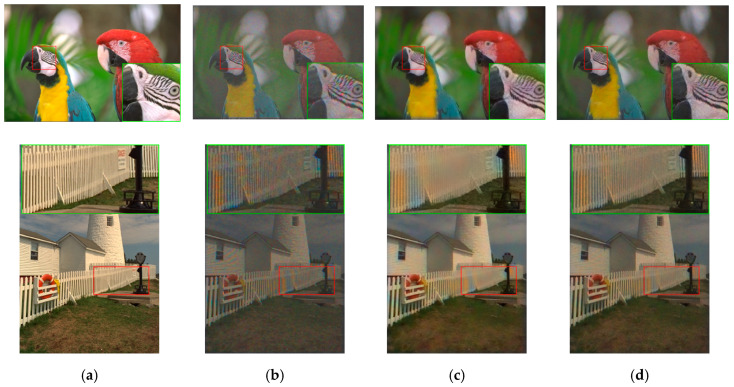
Visual comparison of three high performing demosaicing algorithms at 10 dBs SNR (Poisson noise). The top row is the bird image and the bottom row is the lighthouse image. (**a**) Ground Truth; (**b**) Standard; (**c**) GFPCA; (**d**) F3.

The demosaiced results of Images 1 and 8 are shown in Figure 9. There are color distortion, noise, and contrast issues in the demosaiced images.

It can be observed that, if there is no denoising, all the algorithms have big fluctuations and the demosaiced results are not satisfactory.

● Case 2: Denoising after Demosaicing

In this case, we applied demosaicing first, followed by denoising. The denoising algorithm is BM3D. The denoising was done one band at a time. The F3 method fused the results from Demonet + GFPCA, GFPCA, and GSA. ATMF fused results from Demonet + GFPCA, GFPCA, GSA, PCA, GLP, GS, and PRACS. From Table A4 in Appendix A, the averaged PSNR score of Demonet + GFPCA and GFPCA have much higher scores than the rest. The other methods also yielded around 4 dBs higher scores than those numbers in Table A3.

**Figure 10:**
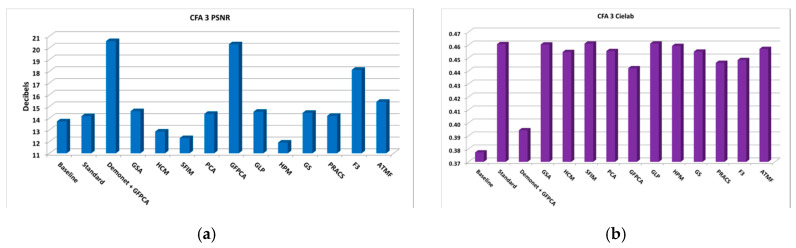

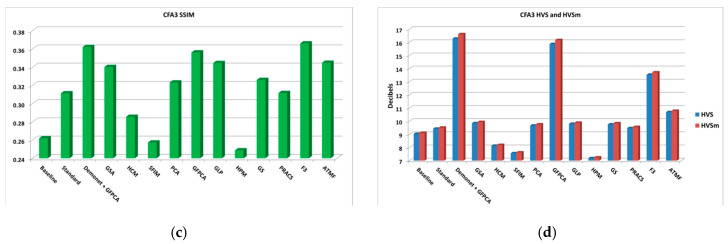
Averaged performance metrics for all the low light images at 10 dBs SNR (Poisson noise). (**a**) PNSR; (**b**) CIELAB; (**c**) SSIM; (**d**) HVS and HVSm.

Figure 10 illustrates the averaged performance metrics, which look much better than those in Figure 8.

**Figure 11:**
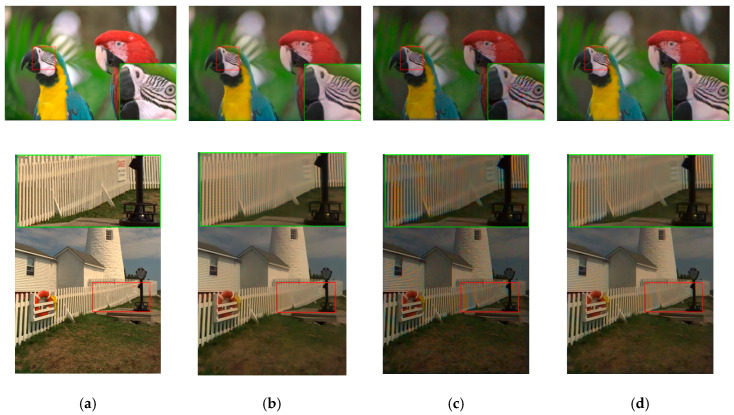
Visual comparison of three high performing demosaicing algorithms at 10 dBs SNR (Poisson noise). The top row is the bird image and the bottom row is the lighthouse image. (**a**) Ground Truth; (**b**) Demonet + GFPCA; (**c**) ATMF; (**d**) F3.

The denoised and demosaiced images of three methods are shown in Figure 11. We observe that the artifacts in Figure 9 have been reduced significantly. Visually speaking, the distortion in the images of Demonet + GFPCA is quite small for the fence area of Image 8.

● Case 3: Denoising before Demosaicing

In this case, we first performed denoising and then demosaicing by pansharpening. The denoising is applied to two places. One is to the luminance image, which is the image after interpolation. The other place is to the reduced resolution color image. Denoising using Akiyama et al. approach [46] is a good alternative and will be a good future direction. The F3 method fused the results from the Standard, Demonet + GFPCA, GSA. ATMF fused the results from Standard, Demonet + GFPCA, GSA, HCM, GFPCA, GLP, and PRACS. From Table A5, we can see that the Demonet + GFPCA algorithm yielded the best averaged PSNR score, which is close to 26 dBs. This is almost 6 dBs better than those numbers in Table A4 and 16 dBs more than those in Table A3. The other metrics in Table A5 are all significantly improved over Table A4. As we will explain later, denoising after demosaicing performs worse than that of before demosaicing.

**Figure 12:**
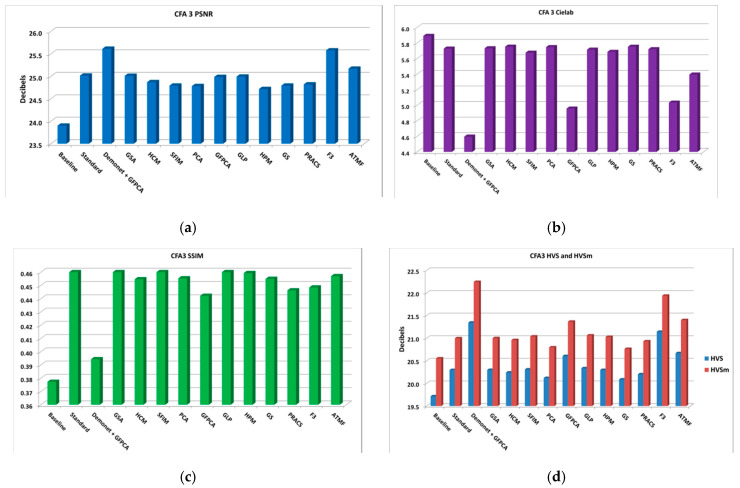
Averaged performance metrics for all the low light images at 10 dBs SNR (Poisson noise). (**a**) PNSR; (**b**) CIELAB; (**c**) SSIM; (**d**) HVS and HVSm.

Figure 12 shows the averaged performance metrics. The metrics are significantly better than those in Figure 8 and Figure 10.

**Figure 13:**
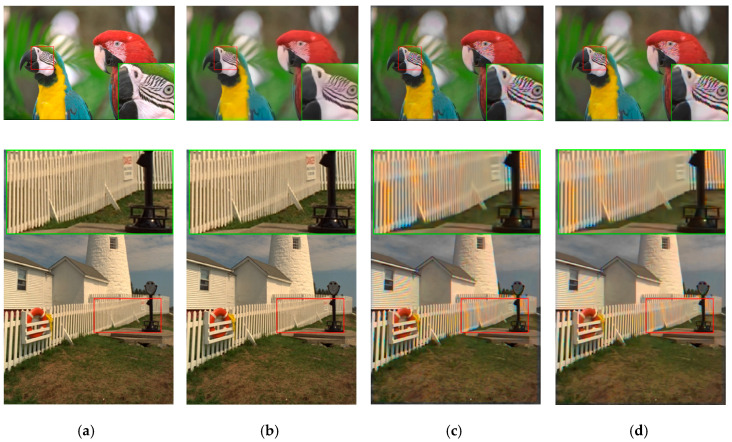
Visual comparison of three high performing demosaicing algorithms at 10 dBs SNR (Poisson noise). The top row is the bird image and the bottom row is the lighthouse image. (**a**) Ground Truth; (**b**) Demonet + GFPCA; (**c**) ATMF; (**d**) F3.

Figure 13 shows the demosaiced images of three methods. We can observe that the demosaiced images have better contrast than those in Figure 11. The Demonet + GFPCA method has less color distortion.

#### 3.2.3. 20 dBs SNR

We have three cases here.

● Case 1: No Denoising (20 dBs SNR)

There are 14 methods. The F3 method fused the three best performing methods: Demonet+GFPCA, GFPCA, and PRACS. ATMF fused the seven best performing methods: Demonet+GFPCA, GFPCA, PRACS, Baseline, GSA, PCA, and GLP. From Table A6 in Appendix A, we can see that the averaged PSNR score of PRACS is the best, which is 21.8 dBs.

**Figure 14:**
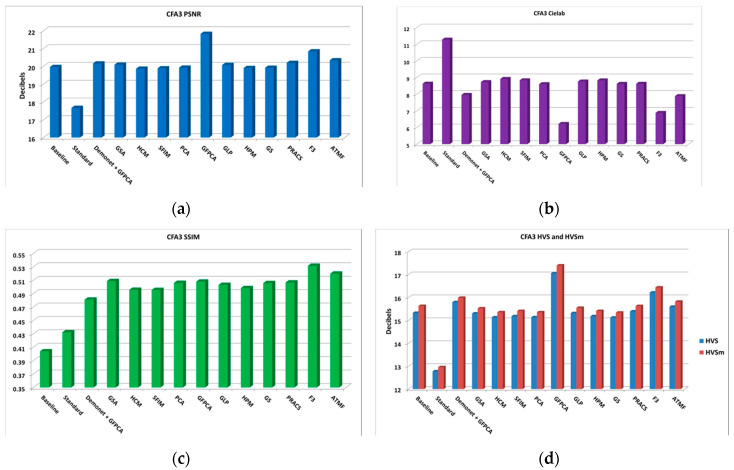
Averaged performance metrics for all the low light images at 20 dBs SNR (Poisson noise). (**a**) PNSR; (**b**) CIELAB; (**c**) SSIM; (**d**) HVS and HVSm.

**Figure 15:**
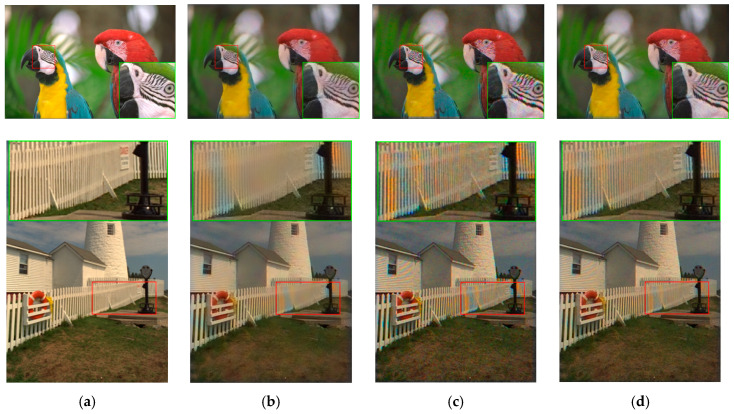
Visual comparison of three high performing demosaicing algorithms at 20 dBs SNR (Poisson noise). The top row is the bird image and the bottom row is the lighthouse image. (**a**) Ground Truth; (**b**) GFPCA; (**c**) ATMF; (**d**) F3.

The average performance metrics are shown in Figure 14. The results are reasonable because there is no denoising capability in demosaicing methods. Figure 15 shows the demosaiced images of three methods: GFPCA, ATMF, and F3. One can easily see some artifacts (color distortion).

● Case 2: Denoising after Demosaicing (20 dBs SNR)

The F3 method performed pixel level fusion using the results of Demonet + GFPCA, GFPCA, and GLP. ATMF fused the results of Demonet + GFPCA, GFPCA, GLP, Standard, GSA, PCA, and GS. From Table A7, we can observe that the Demonet + GFPCA achieved the highest averaged PSNR score of 21.292 dBs. This is better than most of PSNR numbers in Table A6, but only slightly better than the Demonet + GFPCA method (20.573 dBs) in Table A4 (10 dBs SNR case). This clearly shows that denoising has more dramatic impact for low SNR case than with high SNR case. The other metrics in Table A7 are all improved over those numbers in Table A6.

**Figure 16:**
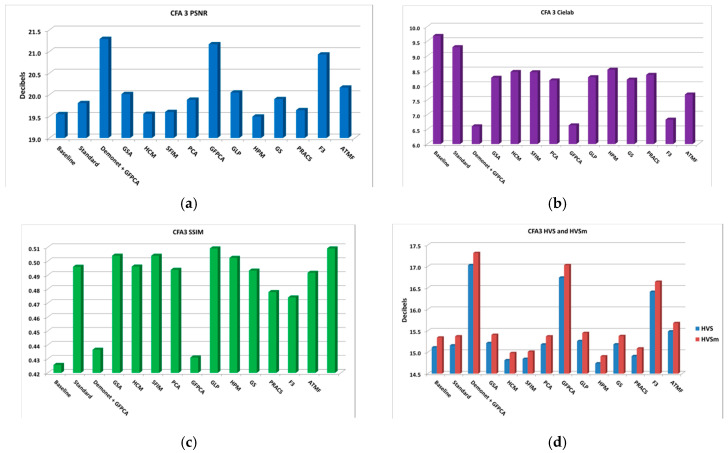
Averaged performance metrics for all the low light images at 20 dBs SNR (Poisson noise). (**a**) PNSR; (**b**) CIELAB; (**c**) SSIM; (**d**) HVS and HVSm.

Figure 16 shows the averaged performance metrics. The numbers are than those in Figure 14.

**Figure 17:**
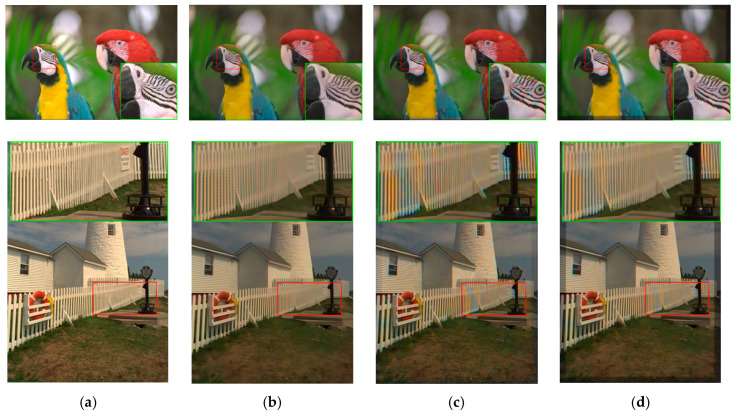
Visual comparison of three high performing demosaicing algorithms at 20 dBs SNR (Poisson noise). The top row is the bird image and the bottom row is the lighthouse image. (**a**) Ground Truth; (**b**) Demonet + GFPCA; (**c**) ATMF; (**d**) F3.

The demosaiced images of three methods are shown in Figure 17. We can see that the artifacts in Figure 17 have been reduced as compared to Figure 15. The color distortions are still noticeable.

● Case 3: Denoising before Demosaicing (20 dBs SNR)

The F3 method fused the results of three best performing methods: Standard, GSA, and GFPCA. ATMF fused the 7 best performing methods: Standard, GSA, GFPCA, HCA, SFIM, GS, and HPM. From Table A8, we can see that F3 yielded 27.07 dBs of PSNR. This is 7 dBs better than the best method in Table A6 and 6 dBs better than the best method in Table A7. The other metrics in Table A8 are all improved over Table A7 quite significantly. This means that the location of denoising is quite critical for improving the overall demosaicing performance.

**Figure 18:**
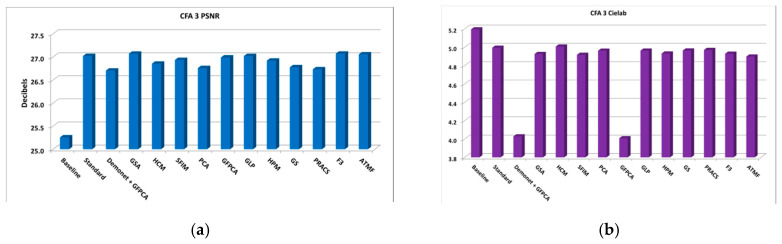

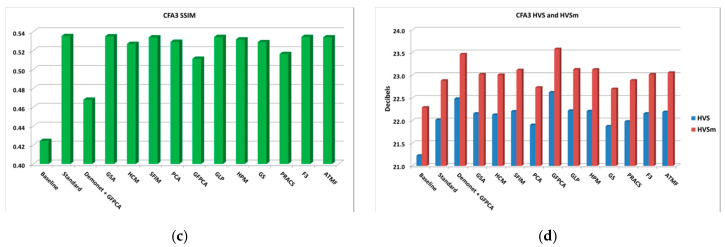
Averaged performance metrics for all the low light images at 20 dBs SNR (Poisson noise). (**a**) PNSR; (**b**) CIELAB; (**c**) SSIM; (**d**) HVS and HVSm.

Figure 18 shows the average performance metrics. The numbers are better than those in Figure 14 and Figure 16.

**Figure 19:**
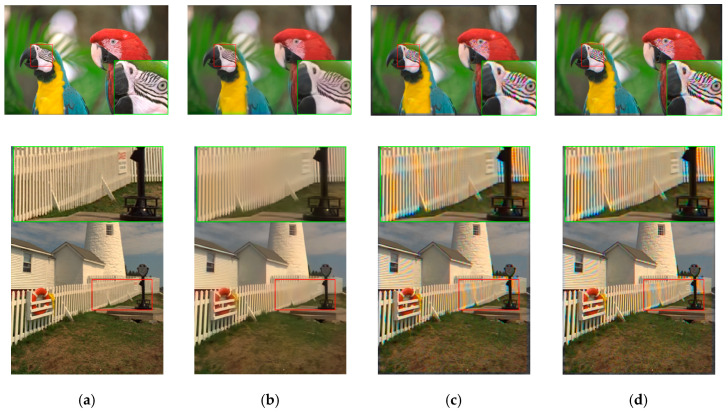
Visual comparison of three high performing demosaicing algorithms at 20 dBs SNR (Poisson noise). The top row is the bird image and the bottom row is the lighthouse image. (**a**) Ground Truth; (**b**) Demonet + GFPCA; (**c**)ATMF; (**d**) F3.

Figure 19 displays the demosaiced images of three selected methods. It is hard to say whether or not the demosaiced images in Figure 19 is better than that of Figure 17 because there are some color distortions.

### 3.3. Comparison of CFAs 1.0, 2.0, and 3.0

As mentioned in Section 1, it will be important to compare the three CFAs and answer the question; which is the best for low lighting images? Given that different algorithms were used in each CFA, selecting the best performing method for each CFA and comparing them against one another will be a good strategy.

We evaluated the following algorithms for CFA 1.0 d in our experiments. Three of them are deep learning based algorithms (Demonet, SEM, and DRL).

- Linear Directional Interpolation and Nonlocal Adaptive Thresholding (LDI-NAT) [16].
- Demosaicnet (Demonet) [30].
- Fusion using 3 best (F3) [32].
- Bilinear [47].
- Malvar–He–Cutler (MHC) [47].
- Directional Linear Minimum Mean Square-Error Estimation (DLMMSE) [48].
- Lu and Tan Interpolation (LT) [49].
- Adaptive Frequency Domain (AFD) [50].
- Alternate Projection (AP). [51].
- Primary-Consistent Soft-Decision (PCSD) [52].
- Alpha Trimmed Mean Filtering (ATMF) [32,53].
- Sequential Energy Minimization (SEM) [54].
- Deep Residual Network (DRL) [55].
- Exploitation of Color Correlation (ECC) [56].
- Minimized-Laplacian Residual Interpolation (MLRI) [57].
- Adaptive Residual Interpolation (ARI) [58].
- Directional Difference Regression (DDR) [59].

### 3.3.1. Noiseless Case (Normal Lighting Conditions)

**Figure 20:**
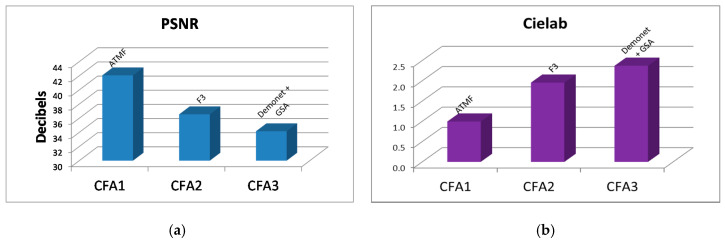

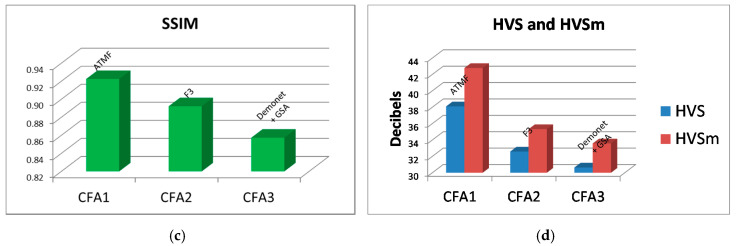
Best against the best comparison between CFAs 1.0, 2.0, and 3.0 in the noiseless case. (**a**) PSNR metrics; (**b**) Cielab metrics; (**c**) SSIM metrics; (**d**) HVS and HVSm metrics.

Here, we compare the performance of CFAs in the noiseless case. The 12 clean Kodak images were used in our study. To save space, we do not provide the image by image performance metrics. Instead, we only summarize the averaged metrics of the different CFAs in Table 1 and Figure 20. In each cell of Table 1, we provide the metric values as well as the name of the best performance method for that metric. One can see that CFA 1.0 is the best in every performance metric, followed by CFA 2.0. CFA 3.0 has the worst performance. We had the same observation for CFA 1.0 and CFA 2.0 in our earlier studies [12].

### 3.3.2. 10 dBs SNR

**Figure 21:**
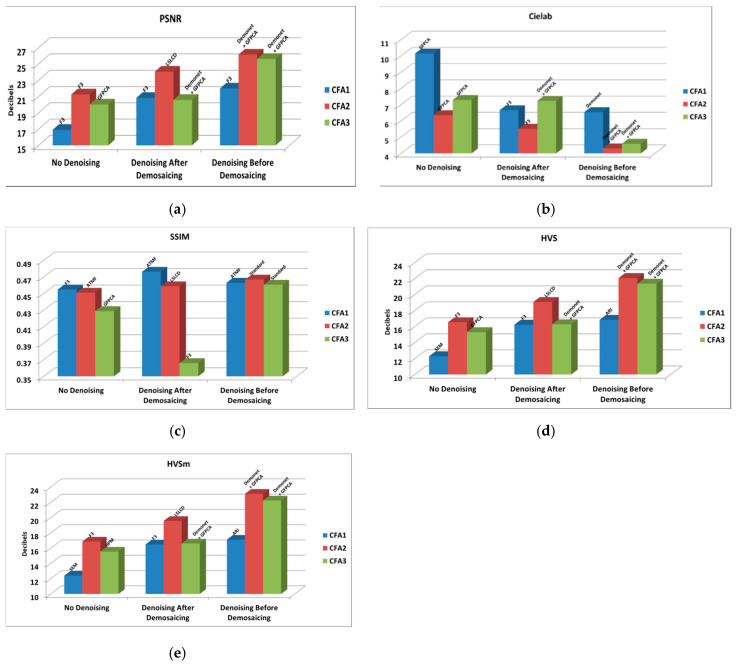
Best against the best comparison between CFAs 1.0, 2.0, and 3.0 with and without denoising at 10 dBs SNR. (**a**) PSNR metrics; (**b**) Cielab metrics; (**c**) SSIM metrics; (**d**) HVS metrics; (**e**) HVSm metrics.

Table 2 and Figure 21 summarize the averaged performance metrics for 10 dBs SNR case in our earlier studies in Section 3.2 for CFA 3.0 and our earlier paper [13] for CFAs 1.0 and 2.0. In Table 2, we include the name of the best performing algorithm. We have the following observations:

- Without denoising, CFAs 1.0, 2.0, and 3.0 have big differences. CFA 2.0 is more than 4 dBs higher than CFA 1.0 and CFA 3.0 is 1.2 dBs lower than CFA 2.0.
- Denoising improves the demosaicing performance independent of the denoising location. For CFA 1.0, the improvement over no denoising is 4 dBs; for CFA 2.0, the improvement is more than 2.7 dBs to 5 dBs; for CFA 3.0, we see 0.57 dBs to 5.6 dBs of improvement in PSNR. We also see dramatic improvements in other metrics,
- Denoising after demosaicing is worse than that of denoising before demosaicing. For CFA 1.0, the improvement is 1.1 dBs with denoising before demosaicing; for CFA 2.0, the improvement is 2.1 dBs with denoising before demosaicing; for CFA 3.0, the improvement is over 5 dBs in PSNR with denoising before demosaicing.
- One important finding is that CFAs 2.0 and 3.0 definitely have advantages over CFA 1.0.
- CFA 2.0 is better than CFA 3.0.

### 3.3.3. 20 dBs SNR

**Figure 22:**
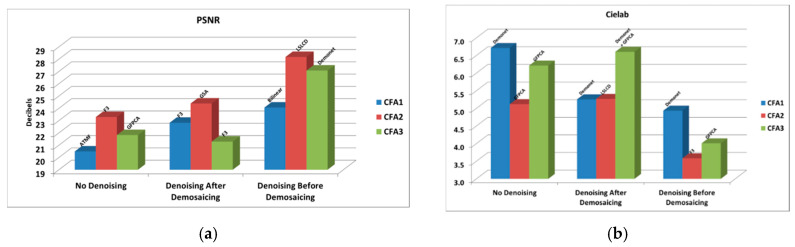

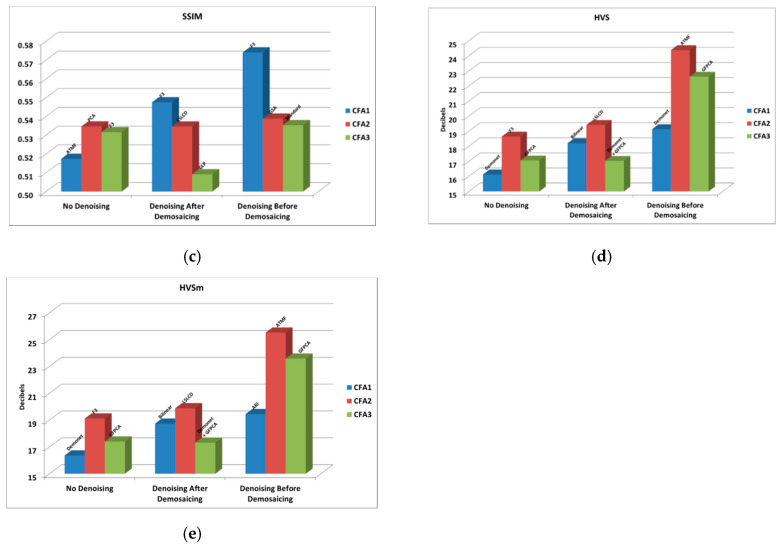
Best against the best comparison between CFAs 1.0, 2.0, and 3.0 with and without denoising at 20 dBs SNR. (**a**) PSNR metrics; (**b**) Cielab metrics; (**c**) SSIM metrics; (**d**) HVS metrics; (**e**) HVSm metrics.

In Table 3 and Figure 22, we summarize the best results for different CFAs under different denoising/demosaicing scenarios presented in earlier sections. Some numbers for CFAs 1.0 and 2.0 in Table 3 came from our earlier paper [13]. The following observations can be drawn:

- Without denoising, CFA 2.0 is the best, followed by CFA 3.0 and CFA 1.0.
- Denoising improves the demosaicing performance in all scenarios. For CFA 1.0, the improvement is over 2 to 4 dBs; for CFA 2.0, the improvement is more than 1 to close to 5 dBs; for CFA 3.0, the improvement is 6 dBs in terms of PSNR. Other metrics have been improved with denoising.
- Denoising after demosaicing is worse than that of denoising before demosaicing. For CFA 1.0, the improvement is 1.2 dBs with denoising before demosaicing; for CFA 2.0, the improvement is close to 4 dBs with denoising before demosaicing; for CFA 3.0, the improvement is close to 6 dBs in PSNR with denoising before demosaicing.
- We observe that CFAs 2.0 and 3.0 definitely have advantages over CFA 1.0.
- CFA 2.0 is better than CFA 3.0.

### 3.4. Discussions

Here, some qualitative analyses/explanations for some of those important findings in Section 3.3.2 and Section 3.3.3 are provided:

● The reason denoising before demosaicing is better that after demosaicing

We explained this phenomenon in our earlier paper [13]. The reason is simply because noise is easier to suppress early than later. Once noise has propagated down the processing pipeline, it is harder to suppress it due to some nonlinear processing modules. For instance, the rectified linear units (ReLu) are nonlinear in some deep learning methods. We have seen similar noise behavior in our active noise suppression project for NASA. In that project [60,61], we noticed that noise near the source was suppressed more effectively than noise far away from the source.

● The reasons why CFA 2.0 and CFA 3.0 are better than CFA 1.0 in low lighting conditions

To the best of our knowledge, we are not aware of any theory explaining why CFA 2.0 and CFA 3.0 have better performance than CFA 1.0. Intuitively, we agree with the inventors of CFA 2.0 that having more white pixels improves the sensitivity of the imager/detector. Here, we offer another explanation.

**Figure 23:**
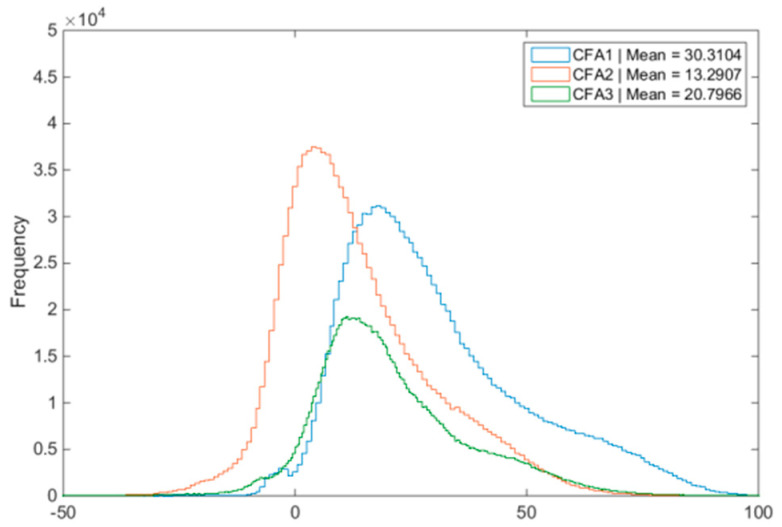
No denoising cases at 10 dBs. Error distributions of the three CFAs.

We use the bird image at 10 dBs condition (Image 1 in Figure 6 of [13]) for explanations. Denoising was not used in the demosaicing process. Figure 23 contains three histograms and the means of the residual images (residual = reference − demosaiced) for CFAs 1.0, 2.0, and 3.0 are also computed. We can see that the histograms of CFA 2.0 and CFA 3.0 are centered near zero whereas the histogram of CFA 1.0 is biased towards to right, meaning that CFA 2.0 and CFA 3.0 are closer to the ground truth, because of their better light sensitivity, than that of CFA 1.0.

● Why CFA 3.0 is NOT better than CFA 2.0 in low lighting conditions

We observe that CFA 3.0 is better than CFA 1.0, but is slightly inferior to CFA 2.0 in dark conditions, which means that having more white pixels can only improve the demosaicing performance to certain extent. Too many white pixels means fewer color pixels and this may degrade the demosaicing performance by having more color distortion. CFA 2.0 is the best compromise between sensitivity and color distortion.

## 4. Conclusions

In this paper, we first introduce a RGBW pattern with 75% of the pixels white, 12.5% of the pixels green, and 6.25% of the pixels red and blue. This is known as the CFA 3.0. Unlike a conventional RGBW pattern with 75% white and the rest pixels are randomly red, green and blue, our pattern is fixed. One key advantage of our pattern is that some of the algorithms for demosaicing CFA 2.0 can be easily adapted to CFA 3.0. Other advantages are also mentioned in Section 1. We then performed extensive experiments to evaluate the CFA 3.0 using clean and emulated low lighting images. After that, we compared the CFAs for various clean and noisy images. Using five objective performance metrics and subjective evaluations, it was observed that, the demosacing performance in CFA 2.0 and CFA 3.0 is indeed better than CFA 1.0. However, more white pixels do not guarantee better performance because CFA 3.0 is slightly worse than CFA 2.0. This is because the color information is less in CFA 3.0, compared to CFA 2.0, causing the loss of color information in the CFA 3.0 case. Denoising further improves the demosaicing performance. In our research, we have experimented with two denoising scenarios: before and after demosaicing. We have seen dramatic performance gain of more than 3 dBs improvement in PSNR for the 10 dBs case when denoising was applied. One important observation is that denoising after demosaicing is worse than denoising before demosaicing. Another observation is that CFA 2.0 with denoising is the best performing algorithm for low lighting conditions.

One potential future direction for research is to investigate different denoising algorithms, such as color BM3D and deep learning based denoising algorithms [62]. Another direction is to investigate joint denoising and demosaicing for CFAs 2.0 and 3.0 directly. Notably, joint denoising and demosaicing has been mostly done for CFA 1.0. The extension of joint denoising and demosaicing to CFAs 2.0 and 3.0 may be non-trivial and needs some further research.

## Figures and Tables

**Table 1:** Comparison of CFAs for different demosaicing method in the noiseless case (normal lighting conditions). Bold numbers indicate the best performing methods in each row.

Metrics	CFA 1.0/Best Algorithm	CFA 2.0/Best Algorithm	CFA 3.0/Best Algorithm
PSNR	**42.068**/ATMF	36.554/F3	34.162/Demonet + GSA
Cielab	**0.996**/ATMF	1.956/F3	2.372/Demonet + GSA
SSIM	**0.922**/ATMF	0.892/F3	0.857/Demonet + GSA
HVS	**38.101**/ATMF	32.590/F3	30.641/Demonet + GSA
HVSm	**42.788**/ATMF	35.325/F3	33.580/Demonet + GSA

**Table 2:** Comparison of CFA patterns for the various demosaicing cases at 10 dBs SNR. Bold numbers indicate the best performing methods in each row.

Metrics	CFA	No Denoising/Best Algorithm	Denoising After Demosaicing/Best Algorithm	Denoising Before Demosaicing/Best Algorithm
PSNR (dB)	1.0	16.889/F3	20.826/F3	21.978/F3
	2.0	**21.249**/F3	**24.050**/LSLCD	**26.141**/Demonet+GFPCA
	3.0	20.018/GFPCA	20.573/Demonet+GFPCA	25.614/Demonet+GFPCA
CIELAB	1.0	10.149/GFPCA	6.664/F3	6.545/Demonet
	2.0	**6.354**/GFPCA	**5.516**/F3	**4.310**/Demonet+GFPCA
	3.0	7.288/GFPCA	7.236/Demonet+GFPCA	4.596/Demonet+GFPCA
SSIM	1.0	**0.455**/F3	**0.476**/ATMF	0.463/ATMF
	2.0	0.451/ATMF	0.459/LSLCD	**0.467**/Standard
	3.0	0.429/GFPCA	0.366/F3	0.461/Standard
HVS (dB)	1.0	12.285/SEM	16.229/F3	16.833/ARI
	2.0	**16.531**/F3	**19.056**/LSLCD	**22.053**/Demonet+GFPCA
	3.0	15.294/GFPCA	16.277/Demonet+GFPCA	21.346/Demonet+GFPCA
HVSm (dB)	1.0	12.403/SEM	16.494/F3	17.116/ARI
	2.0	**16.868**/F3	**19.568**/LSLCD	**23.121**/Demonet+GFPCA
	3.0	15.551/HPM	16.611/Demonet+GFPCA	22.245/Demonet+GFPCA

**Table 3:** Comparison of CFA patterns for the various demosaicing cases at 20 dBs SNR. Bold numbers indicate the best performing methods in each row.

Metrics	CFA	No Denoising/Best Algorithm	Denoising After Demosaicing/Best Algorithm	Denoising Before Demosaicing/Best Algorithm
PSNR (dB)	1.0	20.488/ATMF	22.821/F3	24.059/Bilinear
	2.0	**23.290**/F3	**24.391**/GSA	**28.172**/LSLCD
	3.0	21.821/GFPCA	21.292/F3	27.070/Demonet
CIELAB	1.0	6.713/Demonet	**5.256**/Demonet	4.935/Demonet
	2.0	**5.121**/GFPCA	5.268/LSLCD	**3.584**/F3
	3.0	6.214/GFPCA	6.605/Demonet+GFPCA	4.008/GFPCA
SSIM	1.0	0.517/ATMF	**0.548**/F3	**0.574**/F3
	2.0	**0.535**/PCA	0.535/LSLCD	0.539/GSA
	3.0	0.532/F3	0.509/GLP	0.535/Standard
HVS (dB)	1.0	16.130/Demonet	18.204/Bilinear	19.142/Demonet
	2.0	**18.646**/F3	**19.415**/LSLCD	**24.382**/ATMF
	3.0	17.061/GPCA	17.030/Demonet+GFPCA	22.621/GFPCA
HVSm (dB)	1.0	16.365/Demonet	18.734/Bilinear	19.444/ARI
	2.0	**19.112**/F3	**19.881**/LSLCD	**25.516**/ATMF
	3.0	17.400/GFPCA	17.313/Demonet+GFPCA	23.576/GFPCA

## Appendix A

**Table A1:** Comparison of Gaussian and Poisson noisy images.

SNR	Images with Different Levels of Gaussian Noise (Thermal Noise)	Images with Different Levels of Poisson Noise (Photon Shot Noise)
**20 dB**		
	*σ* = 0.1	*λ*: average number of photons per pixel = 100
**23 dB**		
	*σ* = 0.0707	*λ*: average number of photons per pixel = 200
**26 dB**		
	*σ* = 0.05	*λ*: average number of photons per pixel = 400
**29 dB**		
	*σ* = 0.0354	*λ*: average number of photons per pixel = 800
**32 dB**		
	*σ* = 0.025	*λ*: average number of photons per pixel = 1600
**35 dB**		
	*σ* = 0.0177	*λ*: average number of photons per pixel = 3200
**38 dB**		
	*σ* = 0.0125	*λ*: average number of photons per pixel = 6400

**Table A2:** Performance metrics of 14 algorithms for clean images. Bold numbers indicate the best performing method in each row. Red numbers indicate those methods used in F3 and those red and green numbers indicate those methods used in ATMF. Bold numbers indicate the best performing methods in each row.

Image	Metrics	Baseline	Standard	Demonet + GSA	GSA	HCM	SFIM	PCA	GFPCA	GLP	HPM	GS	PRACS	F3	ATMF	Best Score
Img1	PSNR	31.936	33.894	35.192	34.126	33.526	33.123	33.828	33.158	33.810	33.066	34.130	33.466	36.171	**37.716**	37.716
	Cielab	2.659	2.374	2.250	2.393	2.481	2.699	2.445	2.773	2.429	2.724	2.351	2.453	1.853	**1.560**	1.560
	SSIM	0.739	**0.859**	0.832	0.854	0.831	0.838	0.845	0.806	0.853	0.834	0.858	0.820	0.839	0.857	0.859
	HVS	28.233	27.610	29.428	28.438	28.309	28.453	28.481	27.947	28.353	28.468	28.403	28.407	32.176	**33.731**	33.731
	HVSm	29.767	29.031	31.056	29.881	29.830	29.975	29.934	28.834	29.822	29.983	29.836	29.868	34.065	**36.274**	36.274
Img2	PSNR	26.771	30.585	**34.464**	30.581	30.211	29.918	30.406	30.136	30.089	29.896	30.521	30.230	30.737	32.027	34.464
	Cielab	4.860	3.766	**2.416**	3.803	3.874	3.901	3.891	3.219	3.906	3.904	3.830	3.903	2.826	2.738	2.416
	SSIM	0.685	0.868	**0.885**	0.867	0.856	0.856	0.845	0.823	0.857	0.853	0.850	0.852	0.825	0.858	0.885
	HVS	23.976	24.331	**30.067**	24.486	24.304	24.264	24.726	27.637	24.398	24.228	24.403	24.445	28.565	28.822	30.067
	HVSm	25.515	25.629	**32.745**	25.803	25.665	25.666	26.076	29.840	25.721	25.613	25.703	25.762	31.724	31.215	32.745
Img3	PSNR	30.815	33.017	34.369	32.997	32.156	32.420	32.995	34.055	32.698	32.395	33.037	32.648	35.838	**36.886**	36.886
	Cielab	3.758	3.378	2.836	3.313	3.535	3.432	3.324	2.949	3.345	3.459	3.303	3.398	2.196	**1.907**	1.907
	SSIM	0.786	0.888	0.880	0.884	0.870	0.879	0.877	0.873	0.878	0.873	0.877	0.870	0.883	**0.894**	0.894
	HVS	27.087	27.099	28.646	27.266	27.081	27.211	27.403	29.897	27.192	27.218	27.366	27.221	32.691	**33.472**	33.472
	HVSm	28.861	28.734	30.760	28.928	28.894	28.976	29.065	31.435	28.870	28.973	29.023	28.900	35.473	**36.595**	36.595
Img4	PSNR	22.762	26.980	**30.511**	27.496	27.090	26.808	26.884	26.873	26.762	26.771	26.884	26.933	29.365	29.546	30.511
	Cielab	7.484	5.434	3.775	5.327	5.178	5.314	5.662	4.841	5.664	5.364	5.644	5.371	**3.585**	3.597	3.585
	SSIM	0.752	0.925	**0.946**	0.925	0.919	0.915	0.901	0.891	0.913	0.911	0.903	0.913	0.914	0.925	0.946
	HVS	20.315	20.370	25.427	21.077	21.064	21.160	20.986	24.117	21.095	21.203	20.867	20.918	**26.657**	25.799	26.657
	HVSm	21.997	21.682	27.751	22.476	22.526	22.656	22.377	26.303	22.547	22.706	22.240	22.337	**29.826**	28.261	29.826
Img5	PSNR	30.816	34.107	36.762	33.952	33.686	33.558	33.825	34.541	33.694	33.469	34.132	33.766	36.599	**37.045**	37.045
	Cielab	2.568	2.100	1.593	2.172	2.054	2.107	2.180	1.914	2.132	2.123	2.070	2.136	1.488	**1.459**	1.459
	SSIM	0.668	**0.868**	0.858	0.852	0.859	0.859	0.845	0.798	0.855	0.852	0.859	0.838	0.816	0.827	0.868
	HVS	27.733	27.824	31.730	28.155	28.083	28.083	28.344	30.514	28.154	28.083	28.132	28.146	33.628	**33.670**	33.670
	HVSm	29.444	29.335	34.093	29.707	29.676	29.772	29.912	32.085	29.750	29.775	29.662	29.688	**36.534**	36.333	36.534
Img6	PSNR	27.706	30.874	**33.168**	31.031	30.391	30.382	30.980	31.381	30.647	30.278	30.926	30.601	32.511	33.125	33.168
	Cielab	5.555	4.605	3.393	4.721	4.528	4.464	4.657	3.797	4.619	4.544	4.698	4.575	**3.004**	3.049	3.004
	SSIM	0.711	0.896	**0.909**	0.879	0.877	0.881	0.864	0.848	0.882	0.873	0.860	0.869	0.870	0.890	0.909
	HVS	24.678	24.823	27.010	25.114	24.877	25.031	25.034	27.599	25.159	25.047	25.097	24.967	28.985	**29.193**	29.193
	HVSm	26.353	26.293	28.953	26.606	26.470	26.603	26.520	29.306	26.665	26.611	26.591	26.495	31.643	**31.803**	31.803
Img7	PSNR	30.446	34.517	**38.658**	34.469	34.081	33.701	34.351	33.767	33.917	33.680	34.391	34.183	34.389	35.691	38.658
	Cielab	3.639	2.751	**1.687**	2.773	2.809	2.855	2.799	2.501	2.854	2.857	2.785	2.841	2.141	2.078	1.687
	SSIM	0.731	0.904	**0.920**	0.903	0.896	0.894	0.897	0.853	0.894	0.891	0.897	0.892	0.861	0.894	0.920
	HVS	27.968	28.395	**34.885**	28.409	28.323	28.199	28.497	32.017	28.321	28.159	28.411	28.415	32.584	32.357	34.885
	HVSm	29.538	29.687	**37.761**	29.696	29.661	29.579	29.800	34.461	29.628	29.517	29.706	29.727	36.020	34.752	37.761
Img8	PSNR	26.939	30.748	33.682	31.078	30.419	30.253	30.568	30.319	30.390	30.123	30.670	30.439	33.479	**33.700**	33.700
	Cielab	4.697	3.707	2.707	3.566	3.769	3.704	3.758	3.200	3.746	3.734	3.707	3.812	**2.407**	2.441	2.407
	SSIM	0.733	0.900	**0.910**	0.899	0.885	0.890	0.883	0.860	0.891	0.886	0.888	0.877	0.880	0.890	0.910
	HVS	24.460	24.087	28.285	25.013	24.854	24.969	25.039	28.845	25.004	24.996	24.866	24.883	**31.045**	29.955	31.045
	HVSm	26.141	25.461	30.690	26.453	26.378	26.496	26.473	30.948	26.476	26.519	26.277	26.335	**34.113**	32.146	34.113
Img9	PSNR	29.775	32.268	34.974	32.682	32.117	31.742	32.676	33.783	32.316	31.669	32.668	32.318	35.220	**35.620**	35.620
	Cielab	3.062	2.705	2.017	2.592	2.601	2.911	2.561	2.202	2.669	2.974	2.566	2.595	1.707	**1.683**	1.683
	SSIM	0.508	0.634	**0.643**	0.637	0.623	0.623	0.582	0.615	0.577	0.564	0.582	0.616	0.624	0.631	0.643
	HVS	26.329	26.028	29.823	26.753	26.632	26.808	26.748	30.150	26.823	26.820	26.779	26.621	32.389	**32.588**	32.588
	HVSm	27.955	27.482	31.906	28.234	28.181	28.362	28.228	31.987	28.331	28.365	28.264	28.115	35.387	**35.453**	35.453
Img10	PSNR	27.054	30.354	**33.931**	30.547	29.970	29.885	30.350	31.177	30.014	29.822	30.309	30.118	31.819	32.689	33.931
	Cielab	4.808	3.975	**2.552**	3.930	3.927	3.915	3.991	3.223	4.075	3.940	3.959	3.936	2.625	2.598	2.552
	SSIM	0.687	0.867	**0.868**	0.867	0.856	0.857	0.832	0.802	0.858	0.853	0.855	0.848	0.825	0.848	0.868
	HVS	24.184	24.135	28.936	24.517	24.440	24.459	24.450	28.393	24.508	24.441	24.515	24.458	**29.661**	29.396	29.661
	HVSm	25.796	25.521	31.336	25.928	25.963	25.969	25.867	30.357	25.931	25.936	25.935	25.910	**33.182**	32.163	33.182
Img11	PSNR	29.027	32.011	33.458	32.234	31.703	31.707	32.121	31.682	31.835	31.655	32.143	31.687	33.209	**33.702**	33.702
	Cielab	4.282	3.556	3.004	3.529	3.654	3.606	3.545	3.412	3.628	3.627	3.543	3.605	2.753	**2.686**	2.686
	SSIM	0.722	0.882	**0.894**	0.883	0.866	0.875	0.875	0.840	0.875	0.871	0.876	0.862	0.861	0.877	0.894
	HVS	26.763	26.320	28.596	27.143	27.134	27.175	27.080	28.744	27.177	27.215	27.085	27.089	30.413	**30.445**	30.445
	HVSm	28.417	27.778	30.488	28.626	28.708	28.724	28.548	30.402	28.693	28.762	28.552	28.586	**32.997**	32.899	32.997
Img12	PSNR	25.845	28.451	**30.776**	29.115	28.779	28.769	28.796	29.171	28.807	28.733	28.782	28.712	30.169	29.939	30.776
	Cielab	4.525	3.669	2.741	3.558	3.610	3.621	3.786	3.176	3.707	3.649	3.783	3.620	**2.588**	2.664	2.588
	SSIM	0.770	0.909	**0.925**	0.910	0.903	0.902	0.880	0.883	0.891	0.889	0.880	0.902	0.895	0.900	0.925
	HVS	24.168	23.290	27.638	24.590	24.674	24.658	24.384	27.561	24.584	24.652	24.385	24.521	**28.899**	28.102	28.899
	HVSm	25.807	24.728	29.843	26.100	26.219	26.227	25.842	29.625	26.115	26.211	25.843	26.026	**31.990**	30.705	31.990
Average	PSNR	28.324	31.484	34.162	31.692	31.178	31.022	31.482	31.670	31.248	30.963	31.550	31.258	33.292	33.974	34.162
	Cielab	4.325	3.502	2.581	3.473	3.502	3.544	3.550	3.101	3.564	3.575	3.520	3.520	2.431	**2.372**	2.372
	SSIM	0.708	0.867	0.873	0.863	0.853	0.856	0.844	0.824	0.852	0.846	0.849	0.846	0.841	**0.857**	0.873
	HVS	25.491	25.359	29.206	25.913	25.815	25.872	25.931	28.618	25.897	25.878	25.859	25.841	**30.641**	30.628	30.641
	HVSm	27.132	26.780	31.449	27.370	27.347	27.417	27.387	30.465	27.379	27.414	27.303	27.312	**33.580**	33.217	33.580

**Table A3:** Performance metrics of 14 algorithms at 10 dBs SNR. Bold numbers indicate the best performing methods in each row. Red numbers indicate those methods used in F3 and those red and green numbers indicate those methods used in ATMF.

Image	Metrics	Baseline	Standard	Demonet + GFPCA	GSA	HCM	SFIM	PCA	GFPCA	GLP	HPM	GS	PRACS	F3	ATMF	Best Score
Img1	PSNR	14.003	16.938	16.684	13.050	11.754	9.868	13.250	**19.722**	12.629	9.869	13.115	13.624	18.233	15.051	19.722
	Cielab	16.956	16.675	12.626	19.269	22.974	30.931	18.550	**8.456**	20.411	30.928	18.907	17.823	11.417	14.372	8.456
	SSIM	0.253	0.255	0.240	0.224	0.193	0.134	0.227	**0.337**	0.209	0.136	0.225	0.252	0.303	0.300	0.337
	HVS	8.433	11.451	11.214	7.483	6.179	4.288	7.667	**14.274**	7.064	4.287	7.559	8.049	12.463	9.416	14.274
	HVSm	8.468	11.521	11.274	7.511	6.200	4.302	7.696	**14.370**	7.090	4.302	7.588	8.079	12.533	9.454	14.370
Img2	PSNR	14.138	17.558	15.974	13.947	11.826	11.352	13.448	**20.151**	13.626	11.236	13.988	14.157	18.113	15.236	20.151
	Cielab	13.866	9.674	11.089	14.472	18.717	19.841	15.036	**6.291**	14.997	20.152	14.122	14.104	7.967	11.474	6.291
	SSIM	0.312	0.492	0.456	0.484	0.413	0.397	0.470	0.478	0.475	0.392	0.483	0.471	**0.504**	0.460	0.504
	HVS	9.381	12.136	11.360	9.143	7.045	6.579	8.646	**15.254**	8.832	6.463	9.167	9.353	13.080	10.381	15.254
	HVSm	9.478	12.287	11.456	9.211	7.090	6.620	8.707	**15.536**	8.896	6.503	9.237	9.427	13.238	10.476	15.536
Img3	PSNR	15.795	20.115	18.598	14.278	11.922	10.052	14.552	19.590	14.173	12.771	14.483	15.557	**20.337**	16.976	20.337
	Cielab	14.500	11.636	10.824	17.166	23.320	31.084	16.403	**8.648**	17.410	20.671	16.538	14.949	9.372	12.038	8.648
	SSIM	0.377	0.351	0.383	0.340	0.256	0.160	0.343	**0.437**	0.340	0.298	0.341	0.373	0.417	0.424	0.437
	HVS	10.597	**14.944**	13.688	9.089	6.724	4.846	9.370	14.482	8.988	7.577	9.301	10.364	14.868	11.766	14.944
	HVSm	10.672	**15.165**	13.811	9.141	6.757	4.870	9.427	14.621	9.040	7.615	9.358	10.433	15.031	11.847	15.165
Img4	PSNR	10.088	14.211	14.391	10.306	10.012	10.039	10.178	**18.433**	10.204	10.042	10.319	10.206	15.782	11.786	18.433
	Cielab	24.012	12.199	14.541	24.316	25.080	24.840	23.819	**8.165**	24.683	24.823	23.431	24.210	9.892	17.360	8.165
	SSIM	0.235	0.414	0.483	0.363	0.339	0.343	0.346	**0.554**	0.356	0.344	0.355	0.335	0.518	0.403	0.554
	HVS	5.410	9.014	10.008	5.576	5.291	5.325	5.440	**13.711**	5.490	5.326	5.577	5.483	10.962	7.032	13.711
	HVSm	5.537	9.282	10.236	5.685	5.395	5.429	5.551	**14.282**	5.597	5.431	5.691	5.596	11.285	7.186	14.282
Img5	PSNR	16.916	21.224	17.470	14.163	11.114	11.393	14.693	**22.393**	13.676	9.984	14.249	16.729	20.799	17.823	22.393
	Cielab	10.360	7.301	9.646	14.069	20.711	19.935	13.049	**5.184**	14.891	24.430	13.751	10.656	6.514	8.863	5.184
	SSIM	0.267	0.311	0.296	0.297	0.244	0.251	0.300	**0.371**	0.290	0.213	0.295	0.318	0.350	0.341	0.371
	HVS	12.644	16.258	13.366	9.950	6.905	7.190	10.449	**18.072**	9.470	5.780	10.009	12.476	16.414	13.535	18.072
	HVSm	12.758	16.524	13.464	10.007	6.935	7.222	10.513	**18.329**	9.522	5.804	10.069	12.577	16.615	13.647	18.329
Img6	PSNR	17.726	20.076	18.567	16.210	13.238	14.560	16.054	**22.636**	16.131	10.268	16.374	17.433	21.515	18.790	22.636
	Cielab	13.170	12.025	11.708	15.276	21.065	17.836	14.915	**6.505**	15.380	33.388	14.554	13.681	8.691	10.560	6.505
	SSIM	0.316	0.390	0.380	0.390	0.294	0.345	0.387	**0.442**	0.387	0.107	0.390	0.401	0.439	0.422	0.442
	HVS	13.266	16.102	14.382	11.751	8.822	10.148	11.623	**18.036**	11.690	5.859	11.939	12.933	17.168	14.265	18.036
	HVSm	13.482	16.479	14.576	11.882	8.891	10.238	11.755	**18.506**	11.820	5.903	12.080	13.110	17.554	14.483	18.506
Img7	PSNR	19.036	22.679	18.003	18.817	17.649	18.024	19.394	**22.679**	18.984	18.439	19.216	19.408	22.200	20.065	22.679
	Cielab	9.992	6.905	10.548	10.272	11.420	10.948	9.637	**5.470**	10.122	10.564	9.765	9.826	5.956	8.312	5.470
	SSIM	0.307	0.402	0.341	0.397	0.383	0.384	0.398	0.393	0.394	0.389	0.398	0.400	**0.417**	0.404	0.417
	HVS	14.648	**18.350**	13.822	14.501	13.344	13.730	15.070	18.337	14.669	14.130	14.874	15.037	17.860	15.673	18.350
	HVSm	14.807	**18.701**	13.924	14.657	13.459	13.863	15.248	18.619	14.842	14.279	15.045	15.208	18.106	15.840	18.701
Img8	PSNR	11.581	15.178	17.590	11.734	10.788	10.041	11.971	**20.682**	11.644	10.042	11.765	11.633	17.696	13.332	20.682
	Cielab	21.357	13.557	10.492	21.184	24.372	27.258	20.200	**6.492**	21.492	27.259	20.777	21.450	9.445	16.127	6.492
	SSIM	0.227	0.371	0.400	0.322	0.271	0.230	0.327	**0.452**	0.319	0.230	0.319	0.299	0.437	0.357	0.452
	HVS	6.592	9.667	12.841	6.723	5.785	5.041	6.956	**15.729**	6.640	5.041	6.751	6.627	12.521	8.260	15.729
	HVSm	6.651	9.772	12.981	6.772	5.826	5.078	7.008	**16.030**	6.688	5.078	6.802	6.678	12.670	8.330	16.030
Img9	PSNR	10.053	11.090	14.208	10.068	10.062	10.064	10.027	**17.474**	10.071	10.065	10.026	10.066	14.001	11.048	17.474
	Cielab	17.176	15.676	10.481	17.232	17.270	17.509	17.109	**7.037**	17.242	17.513	17.104	17.207	10.216	14.392	7.037
	SSIM	0.194	0.251	0.292	0.266	0.259	0.258	0.267	**0.314**	0.265	0.258	0.267	0.257	0.308	0.281	0.314
	HVS	5.504	6.472	9.733	5.517	5.514	5.524	5.479	**12.883**	5.523	5.524	5.479	5.513	9.405	6.479	12.883
	HVSm	5.530	6.505	9.776	5.540	5.537	5.547	5.502	**12.965**	5.546	5.547	5.502	5.537	9.448	6.506	12.965
Img10	PSNR	13.625	19.239	17.815	13.493	11.736	12.040	13.348	19.483	13.289	12.158	13.645	13.876	**20.142**	15.465	20.142
	Cielab	16.194	9.271	10.115	16.750	20.876	19.974	16.644	7.487	17.188	19.669	16.111	15.937	**7.392**	12.291	7.392
	SSIM	0.264	0.344	0.395	0.354	0.294	0.309	0.350	**0.433**	0.349	0.314	0.354	0.349	0.422	0.387	0.433
	HVS	9.662	15.279	14.100	9.501	7.766	8.077	9.370	15.398	9.312	8.194	9.659	9.883	**16.333**	11.467	16.333
	HVSm	9.769	15.657	14.267	9.584	7.825	8.138	9.455	15.663	9.391	8.256	9.748	9.977	**16.678**	11.589	16.678
Img11	PSNR	14.825	**19.458**	15.178	14.240	11.081	10.053	14.255	18.317	14.172	10.053	14.349	14.901	18.144	15.610	19.458
	Cielab	14.903	10.783	14.288	16.157	24.628	28.967	15.864	**9.108**	16.304	28.966	15.687	14.906	9.864	13.027	9.108
	SSIM	0.321	0.421	0.365	0.400	0.270	0.209	0.397	0.425	0.397	0.210	0.399	0.407	**0.438**	0.414	0.438
	HVS	9.652	**14.350**	10.008	9.035	5.862	4.830	9.066	13.151	8.974	4.830	9.160	9.697	12.659	10.372	14.350
	HVSm	9.717	**14.531**	10.066	9.085	5.888	4.852	9.117	13.272	9.024	4.852	9.212	9.756	12.766	10.437	14.531
Img12	PSNR	12.443	16.404	16.748	12.545	11.357	11.462	12.647	**18.653**	12.397	11.704	12.579	12.529	17.472	13.971	18.653
	Cielab	19.343	9.549	11.380	19.458	23.079	22.681	18.746	**8.613**	19.897	21.887	18.927	19.410	8.795	14.948	8.613
	SSIM	0.284	0.435	0.457	0.379	0.307	0.317	0.379	**0.511**	0.373	0.333	0.376	0.368	0.497	0.422	0.511
	HVS	7.785	11.524	12.328	7.826	6.645	6.752	7.934	**14.199**	7.686	6.994	7.866	7.820	12.814	9.296	14.199
	HVSm	7.861	11.682	12.455	7.888	6.696	6.803	7.999	**14.415**	7.746	7.048	7.930	7.885	12.976	9.382	14.415
Average	PSNR	14.186	17.847	16.769	13.571	11.878	11.579	13.651	**20.018**	13.416	11.386	13.676	14.176	18.703	15.429	20.018
	Cielab	15.986	11.271	11.478	17.135	21.126	22.650	16.664	**7.288**	17.501	23.354	16.639	16.180	8.793	12.814	7.288
	SSIM	0.280	0.370	0.374	0.351	0.294	0.278	0.349	**0.429**	0.346	0.269	0.350	0.353	0.421	0.385	0.429
	HVS	9.465	12.962	12.237	8.841	7.157	6.861	8.922	**15.294**	8.695	6.667	8.945	9.436	13.879	10.662	15.294
	HVSm	9.561	13.176	12.357	8.913	7.208	6.914	8.998	**15.551**	8.767	6.718	9.022	9.522	14.075	10.765	15.551

**Table A4:** Performance metrics of 14 algorithms at 10 dBs SNR (Poisson noise). Bold numbers indicate the best performing methods in each row. Bold numbers indicate the best performing methods in each row. Red numbers indicate those methods used in F3 and those red and green numbers indicate those methods used in ATMF.

Image	Metrics	Baseline	Standard	Demonet + GFPCA	GSA	HCM	SFIM	PCA	GFPCA	GLP	HPM	GS	PRACS	F3	ATMF	Best Score
Img1	PSNR	13.054	13.763	20.419	13.821	12.359	9.859	13.482	**22.570**	13.670	9.902	13.537	13.451	18.180	14.655	22.570
	Cielab	18.647	16.976	7.829	16.870	20.590	30.676	17.467	**6.312**	17.244	30.447	17.380	17.691	9.786	15.006	6.312
	SSIM	0.263	0.298	0.378	0.300	0.251	0.135	0.287	**0.393**	0.302	0.138	0.289	0.282	0.373	0.320	0.393
	HVS	7.477	8.178	14.962	8.233	6.773	4.272	7.917	**17.219**	8.081	4.314	7.977	7.867	12.654	9.077	17.219
	HVSm	7.500	8.204	15.040	8.258	6.792	4.286	7.941	**17.350**	8.105	4.327	8.002	7.891	12.706	9.106	17.350
Img2	PSNR	14.691	14.245	**22.164**	14.243	12.354	11.945	13.758	20.236	13.946	11.807	14.358	14.446	18.371	15.213	22.164
	Cielab	12.513	13.303	**5.076**	13.363	16.937	17.877	13.987	6.190	13.864	18.219	12.983	13.015	7.658	11.564	5.076
	SSIM	0.298	**0.402**	0.359	0.401	0.340	0.330	0.379	0.341	0.398	0.324	0.394	0.377	0.380	0.389	0.402
	HVS	9.984	9.449	**18.107**	9.457	7.586	7.176	9.009	15.742	9.165	7.039	9.597	9.681	13.746	10.449	18.107
	HVSm	10.074	9.523	**18.542**	9.527	7.637	7.222	9.074	16.024	9.231	7.084	9.670	9.756	13.916	10.535	18.542
Img3	PSNR	14.199	15.322	**23.477**	15.261	12.855	10.311	15.116	23.322	15.238	13.884	15.161	14.948	19.909	16.347	23.477
	Cielab	16.573	14.459	6.137	14.560	19.881	29.327	14.695	**6.050**	14.634	17.259	14.621	15.147	8.333	12.554	6.050
	SSIM	0.356	0.417	**0.510**	0.417	0.322	0.170	0.402	0.506	0.423	0.376	0.404	0.396	0.495	0.441	0.510
	HVS	9.001	10.106	**18.703**	10.043	7.648	5.098	9.943	18.380	10.015	8.666	9.987	9.742	14.811	11.159	18.703
	HVSm	9.048	10.160	**18.944**	10.096	7.684	5.124	9.997	18.636	10.067	8.708	10.041	9.793	14.930	11.224	18.944
Img4	PSNR	10.189	6.944	**18.732**	10.505	10.126	10.284	10.372	17.658	10.450	10.170	10.526	10.305	14.975	11.560	18.732
	Cielab	23.401	40.595	**8.485**	23.191	24.114	23.606	22.890	9.357	23.442	23.937	22.463	23.334	12.094	19.078	8.485
	SSIM	0.269	0.069	**0.568**	0.374	0.346	0.359	0.354	0.549	0.370	0.350	0.364	0.336	0.527	0.419	0.568
	HVS	5.533	2.237	**14.723**	5.776	5.406	5.555	5.684	13.327	5.725	5.442	5.836	5.596	10.439	6.860	14.723
	HVSm	5.646	2.304	**15.210**	5.885	5.508	5.660	5.794	13.726	5.833	5.545	5.947	5.704	10.666	6.989	15.210
Img5	PSNR	11.887	14.896	**20.735**	14.899	11.747	11.955	14.294	20.445	15.314	10.399	14.592	12.938	18.303	15.653	20.735
	Cielab	18.195	12.349	**6.012**	12.384	18.598	18.101	13.195	6.191	11.796	22.657	12.719	15.824	7.919	11.070	6.012
	SSIM	0.191	0.285	0.290	0.287	0.220	0.231	0.269	0.289	**0.297**	0.182	0.272	0.240	0.297	0.288	0.297
	HVS	7.681	10.661	**16.575**	10.667	7.534	7.740	10.088	16.263	11.080	6.189	10.381	8.722	14.099	11.433	16.575
	HVSm	7.716	10.715	**16.728**	10.718	7.566	7.771	10.135	16.415	11.135	6.214	10.432	8.760	14.194	11.492	16.728
Img6	PSNR	17.145	18.931	22.062	19.160	15.673	17.544	18.980	**22.510**	19.092	10.482	18.896	18.884	21.256	19.642	22.510
	Cielab	12.344	10.555	6.888	10.281	14.512	11.813	10.388	**6.350**	10.408	31.443	10.518	10.501	7.320	9.252	6.350
	SSIM	0.270	0.362	0.291	0.368	0.293	0.345	0.349	0.297	**0.373**	0.071	0.344	0.336	0.332	0.351	0.373
	HVS	12.763	14.422	17.955	14.627	11.252	13.084	14.572	**18.354**	14.566	6.073	14.510	14.415	16.945	15.192	18.354
	HVSm	12.922	14.634	18.374	14.855	11.360	13.241	14.799	**18.840**	14.787	6.120	14.734	14.636	17.291	15.441	18.840
Img7	PSNR	20.804	21.559	**28.587**	21.513	20.219	20.428	21.585	27.927	21.191	20.557	21.383	21.165	26.306	22.678	28.587
	Cielab	7.713	7.211	**3.255**	7.257	8.033	7.914	7.111	3.511	7.470	7.836	7.204	7.501	4.089	6.180	3.255
	SSIM	0.310	0.406	0.332	0.406	0.395	0.401	0.393	0.325	**0.407**	0.400	0.392	0.379	0.372	0.392	0.407
	HVS	16.526	17.130	**25.750**	17.087	15.847	16.036	17.276	24.619	16.779	16.165	17.059	16.797	22.404	18.355	25.750
	HVSm	16.718	17.331	**27.238**	17.284	15.993	16.188	17.483	25.799	16.962	16.322	17.257	16.985	23.043	18.613	27.238
Img8	PSNR	12.238	12.295	**19.205**	12.285	11.176	10.361	11.770	18.268	12.361	10.397	11.970	12.300	16.076	13.164	19.205
	Cielab	19.157	19.098	**7.794**	19.126	22.505	25.510	20.395	8.565	18.963	25.372	19.810	19.081	11.231	16.677	7.794
	SSIM	0.234	0.285	**0.347**	0.284	0.230	0.190	0.250	0.331	0.294	0.193	0.259	0.268	0.334	0.296	0.347
	HVS	7.269	7.279	**14.530**	7.272	6.172	5.354	6.784	13.440	7.347	5.390	6.983	7.301	11.161	8.171	14.530
	HVSm	7.326	7.333	**14.705**	7.325	6.216	5.394	6.834	13.596	7.400	5.429	7.034	7.355	11.260	8.233	14.705
Img9	PSNR	9.974	9.187	**17.493**	10.204	10.298	10.177	10.155	16.885	10.148	10.155	10.166	10.072	14.214	11.165	17.493
	Cielab	17.009	18.860	**6.902**	16.519	16.314	16.811	16.487	7.365	16.669	16.869	16.460	16.784	9.822	14.392	6.902
	SSIM	0.187	0.206	**0.273**	0.227	0.226	0.226	0.226	0.263	0.230	0.225	0.226	0.213	0.255	0.236	0.273
	HVS	5.434	4.639	**13.022**	5.652	5.748	5.630	5.618	12.352	5.595	5.607	5.629	5.523	9.679	6.619	13.022
	HVSm	5.454	4.656	**13.084**	5.672	5.768	5.650	5.638	12.413	5.615	5.627	5.649	5.543	9.716	6.642	13.084
Img10	PSNR	13.846	14.159	19.044	14.502	12.749	12.486	14.401	**20.421**	14.368	12.863	14.432	14.260	17.685	15.207	20.421
	Cielab	15.269	14.796	7.789	14.230	17.673	18.275	14.128	**6.745**	14.524	17.390	14.117	14.555	9.090	12.623	6.745
	SSIM	0.257	0.323	0.326	0.334	0.279	0.278	0.321	0.335	0.337	0.291	0.319	0.304	**0.342**	0.332	0.342
	HVS	9.910	10.169	15.340	10.503	8.780	8.511	10.451	**16.645**	10.372	8.887	10.487	10.286	13.806	11.251	16.645
	HVSm	10.004	10.262	15.568	10.605	8.851	8.580	10.553	**16.970**	10.469	8.960	10.590	10.385	13.983	11.364	16.970
Img11	PSNR	14.151	15.449	**17.674**	15.399	12.933	10.055	15.312	16.688	15.444	10.137	15.307	14.756	16.562	15.622	17.674
	Cielab	15.534	13.262	**9.881**	13.350	18.342	28.579	13.321	10.893	13.312	28.180	13.329	14.411	11.154	12.713	9.881
	SSIM	0.251	0.331	0.254	0.332	0.255	0.128	0.317	0.241	**0.344**	0.133	0.316	0.294	0.286	0.315	0.344
	HVS	8.972	10.247	**12.554**	10.196	7.724	4.832	10.161	11.590	10.241	4.914	10.156	9.562	11.413	10.460	12.554
	HVSm	9.023	10.310	**12.657**	10.257	7.762	4.856	10.224	11.678	10.302	4.938	10.218	9.617	11.493	10.525	12.657
Img12	PSNR	12.461	13.288	**17.288**	13.318	11.758	12.120	13.142	16.750	13.281	12.222	13.095	12.842	15.660	13.835	17.288
	Cielab	18.954	16.971	**10.784**	16.903	21.173	20.054	17.016	11.564	17.035	19.758	17.130	18.012	12.756	15.710	10.784
	SSIM	0.257	0.350	**0.416**	0.352	0.268	0.294	0.332	0.404	0.357	0.300	0.330	0.314	0.400	0.360	0.416
	HVS	7.811	8.578	**13.113**	8.609	7.053	7.410	8.465	12.450	8.572	7.511	8.418	8.150	11.206	9.189	13.113
	HVSm	7.880	8.651	**13.249**	8.683	7.110	7.470	8.539	12.580	8.645	7.573	8.491	8.219	11.309	9.268	13.249
Average	PSNR	13.720	14.170	**20.573**	14.593	12.854	12.294	14.364	20.306	14.542	11.914	14.452	14.197	18.125	15.395	20.573
	Cielab	16.276	16.536	**7.236**	14.836	18.223	20.712	15.090	7.424	14.947	21.614	14.894	15.488	9.271	13.068	7.236
	SSIM	0.262	0.311	0.362	0.340	0.285	0.257	0.323	0.356	0.344	0.249	0.326	0.312	**0.366**	0.345	0.366
	HVS	9.030	9.425	**16.278**	9.843	8.127	7.558	9.664	15.865	9.795	7.183	9.752	9.470	13.530	10.685	16.278
	HVSm	9.109	9.507	**16.612**	9.931	8.187	7.620	9.751	16.169	9.879	7.237	9.839	9.554	13.709	10.786	16.612

**Table A5:** Performance metrics of 14 algorithms at 10 dBs SNR (Poisson noise). Bold numbers indicate the best performing methods in each row. Red numbers indicate those methods used in F3 and those red and green numbers indicate those methods used in ATMF.

Image	Metrics	Baseline	Standard	Demonet + GFPCA	GSA	HCM	SFIM	PCA	GFPCA	GLP	HPM	GS	PRACS	F3	ATMF	Best Score
Img1	PSNR	21.413	21.576	**25.348**	21.575	21.542	20.811	21.472	21.408	21.620	21.253	21.478	21.520	22.802	21.636	25.348
	Cielab	7.003	6.968	**5.285**	6.970	7.012	7.027	6.862	7.185	6.977	7.032	6.964	6.975	6.231	6.924	5.285
	SSIM	0.408	0.427	0.384	0.427	0.420	0.430	0.425	0.414	**0.431**	0.429	0.425	0.420	0.422	0.425	0.431
	HVS	15.983	16.049	**19.960**	16.051	16.038	16.076	15.861	15.917	16.089	16.082	15.972	16.033	17.300	16.136	19.960
	HVSm	16.121	16.163	**20.235**	16.165	16.154	16.188	15.969	16.029	16.199	16.194	16.085	16.154	17.442	16.248	20.235
Img2	PSNR	23.326	24.749	**25.720**	24.728	24.646	24.671	24.473	24.481	24.713	24.679	24.520	24.519	25.430	24.828	25.720
	Cielab	5.144	4.796	**3.706**	4.827	4.907	4.848	4.857	4.195	4.835	4.850	4.845	4.887	4.203	4.580	3.706
	SSIM	0.355	**0.505**	0.399	0.504	0.499	0.503	0.500	0.451	0.504	0.502	0.499	0.483	0.480	0.493	0.505
	HVS	19.126	19.932	**21.434**	19.986	19.917	20.019	19.866	19.865	20.067	20.022	19.743	19.887	20.883	20.212	21.434
	HVSm	20.004	20.643	**22.707**	20.682	20.629	20.737	20.530	20.667	20.784	20.744	20.409	20.614	21.781	20.957	22.707
Img3	PSNR	28.287	29.290	28.498	29.287	29.044	29.157	29.201	**29.841**	29.176	29.127	29.201	29.059	29.503	29.557	29.841
	Cielab	4.998	4.908	4.659	4.910	5.039	4.775	4.950	**4.376**	4.857	4.778	4.946	4.928	4.574	4.679	4.376
	SSIM	0.535	0.566	0.523	0.566	0.558	0.566	0.562	**0.574**	0.565	0.564	0.562	0.558	0.563	0.567	0.574
	HVS	24.189	24.566	24.453	24.569	24.490	24.333	24.580	**25.612**	24.282	24.286	24.521	24.493	25.242	25.136	25.612
	HVSm	25.468	25.701	25.513	25.702	25.656	25.489	25.697	**26.834**	25.432	25.442	25.633	25.646	26.423	26.319	26.834
Img4	PSNR	18.115	19.491	**20.725**	19.491	19.315	19.455	19.081	19.584	19.492	19.472	19.087	19.266	20.295	19.810	20.725
	Cielab	12.058	11.913	**6.315**	11.904	11.702	11.465	11.690	7.298	11.894	11.458	11.702	11.636	8.996	10.120	6.315
	SSIM	0.442	0.621	0.594	0.621	0.606	0.615	0.608	0.606	0.617	0.614	0.608	0.593	**0.634**	0.625	0.634
	HVS	13.571	14.226	**16.202**	14.231	14.160	14.318	13.898	14.798	14.351	14.325	13.837	14.103	15.352	14.783	16.202
	HVSm	14.298	14.799	**17.053**	14.803	14.756	14.922	14.446	15.441	14.950	14.934	14.385	14.711	16.005	15.384	17.053
Img5	PSNR	27.738	29.195	27.871	29.189	28.964	29.083	28.794	29.213	29.180	29.066	28.857	28.935	29.113	**29.348**	29.348
	Cielab	3.564	3.447	3.578	3.437	3.424	3.401	3.563	**3.287**	3.402	3.403	3.527	3.429	3.328	3.306	3.287
	SSIM	0.309	0.362	0.312	**0.362**	0.359	0.362	0.358	0.351	0.361	0.360	0.358	0.354	0.353	0.359	0.362
	HVS	23.941	24.909	23.775	24.937	24.747	24.935	24.542	25.109	25.054	24.898	24.476	24.789	25.122	**25.329**	25.329
	HVSm	25.218	25.996	24.688	26.018	25.873	26.095	25.513	26.217	26.191	26.065	25.466	25.907	26.190	**26.446**	26.446
Img6	PSNR	22.216	22.790	**26.248**	22.790	22.729	22.809	22.608	22.670	22.830	22.812	22.602	22.677	24.363	22.876	26.248
	Cielab	6.988	6.826	**5.124**	6.835	6.870	6.736	6.977	6.269	6.787	6.744	6.993	6.829	5.794	6.575	5.124
	SSIM	0.315	0.396	0.334	0.396	0.391	**0.398**	0.391	0.375	0.398	0.397	0.389	0.379	0.383	0.392	0.398
	HVS	18.162	18.526	**21.866**	18.496	18.529	18.604	18.374	18.394	18.590	18.612	18.346	18.421	20.089	18.674	21.866
	HVSm	18.748	19.018	**23.111**	18.997	19.016	19.100	18.872	18.906	19.093	19.110	18.837	18.946	20.811	19.184	23.111
Img7	PSNR	26.556	27.545	26.766	27.552	27.510	27.475	**27.623**	27.511	27.484	27.459	27.620	27.449	27.506	27.603	27.623
	Cielab	4.571	4.375	4.284	4.381	4.416	4.403	4.369	**4.074**	4.389	4.407	4.365	4.411	4.219	4.278	4.074
	SSIM	0.370	0.471	0.359	**0.471**	0.468	0.471	0.467	0.439	0.470	0.469	0.467	0.460	0.447	0.462	0.471
	HVS	22.670	23.285	22.958	23.297	23.293	23.285	23.434	23.481	23.284	23.271	23.377	23.236	**23.509**	23.474	23.509
	HVSm	23.537	24.040	23.825	24.050	24.051	24.052	24.212	**24.332**	24.051	24.040	24.151	24.005	24.327	24.263	24.332
Img8	PSNR	24.878	27.449	26.633	27.431	27.113	27.169	26.931	26.854	27.281	27.118	26.971	26.997	**28.302**	27.760	28.302
	Cielab	4.656	4.376	3.770	4.382	4.438	4.329	4.474	**3.727**	4.342	4.337	4.466	4.432	3.782	4.087	3.727
	SSIM	0.405	0.497	0.395	0.496	0.491	0.497	0.487	0.466	**0.498**	0.495	0.488	0.480	0.476	0.491	0.498
	HVS	20.886	22.153	22.682	22.182	21.976	22.040	21.972	22.889	22.135	21.941	21.771	21.992	**23.938**	23.161	23.938
	HVSm	22.170	23.285	23.983	23.308	23.147	23.251	23.017	24.227	23.342	23.156	22.805	23.150	**25.448**	24.424	25.448
Img9	PSNR	26.090	27.195	25.893	27.199	26.962	26.001	26.958	**27.534**	27.185	24.740	26.949	27.029	27.094	27.444	27.534
	Cielab	3.906	3.830	3.691	3.832	3.803	3.916	3.871	**3.323**	3.791	4.015	3.876	3.778	3.544	3.556	3.323
	SSIM	0.254	0.304	**0.312**	0.304	0.299	0.299	0.303	0.306	0.298	0.293	0.303	0.295	0.303	0.305	0.312
	HVS	21.795	22.290	21.378	22.289	22.173	22.310	22.051	**23.058**	22.376	22.321	22.072	22.179	22.485	22.803	23.058
	HVSm	22.649	22.955	21.837	22.956	22.871	23.013	22.689	**23.693**	23.065	23.027	22.708	22.879	23.059	23.443	23.693
Img10	PSNR	23.651	24.859	24.761	24.856	24.734	24.856	24.592	24.947	24.888	24.850	24.581	24.601	**25.080**	24.975	25.080
	Cielab	5.637	5.408	**4.592**	5.413	5.452	5.353	5.489	4.599	5.410	5.358	5.478	5.434	4.877	5.087	4.592
	SSIM	0.339	0.421	0.359	0.421	0.415	**0.424**	0.417	0.407	0.423	0.422	0.414	0.404	0.408	0.417	0.424
	HVS	20.205	20.975	21.145	20.916	20.967	21.069	20.602	21.417	21.053	21.074	20.672	20.760	**21.438**	21.311	21.438
	HVSm	21.280	21.863	22.176	21.821	21.856	21.981	21.482	**22.415**	21.977	21.991	21.548	21.712	22.405	22.246	22.415
Img11	PSNR	23.264	23.807	**25.878**	23.805	23.747	23.817	23.642	23.617	23.833	23.817	23.639	23.696	24.728	23.853	25.878
	Cielab	5.797	5.695	**4.920**	5.699	5.736	5.680	5.713	5.547	5.687	5.681	5.710	5.696	5.205	5.579	4.920
	SSIM	0.344	0.407	0.311	0.407	0.403	0.413	0.402	0.387	**0.414**	0.413	0.402	0.392	0.383	0.403	0.414
	HVS	18.656	18.887	**21.200**	18.887	18.885	18.936	18.741	18.771	18.946	18.943	18.736	18.842	19.899	19.013	21.200
	HVSm	19.116	19.278	**22.031**	19.277	19.278	19.328	19.123	19.177	19.339	19.337	19.118	19.248	20.405	19.407	22.031
Img12	PSNR	21.292	22.242	**23.025**	22.234	22.129	22.209	22.003	22.171	22.248	22.213	22.006	22.086	22.711	22.350	23.025
	Cielab	6.368	6.190	**5.230**	6.195	6.229	6.167	6.145	5.601	6.198	6.168	6.145	6.203	5.646	5.958	5.230
	SSIM	0.451	0.551	0.450	0.551	0.547	**0.553**	0.543	0.528	0.553	0.552	0.543	0.538	0.528	0.546	0.553
	HVS	17.419	17.757	**19.104**	17.750	17.733	17.793	17.542	17.964	17.820	17.804	17.539	17.705	18.449	18.019	19.104
	HVSm	18.029	18.253	**19.786**	18.245	18.234	18.306	18.021	18.462	18.326	18.318	18.018	18.217	18.984	18.509	19.786
Average	PSNR	23.902	25.016	**25.614**	25.011	24.870	24.793	24.782	24.986	24.994	24.717	24.793	24.820	25.577	25.170	25.614
	Cielab	5.891	5.728	**4.596**	5.732	5.752	5.675	5.747	4.957	5.714	5.686	5.752	5.720	5.033	5.394	4.596
	SSIM	0.377	**0.461**	0.394	0.460	0.455	0.461	0.455	0.442	0.461	0.459	0.455	0.446	0.448	0.457	0.461
	HVS	19.717	20.296	**21.346**	20.299	20.242	20.310	20.122	20.606	20.337	20.298	20.088	20.203	21.142	20.671	21.346
	HVSm	20.553	20.999	**22.245**	21.002	20.960	21.039	20.798	21.367	21.062	21.030	20.764	20.932	21.940	21.403	22.245

**Table A6:** Performance metrics of 14 algorithms at 20 dBs SNR. Bold numbers indicate the best performing methods in each row. Red numbers indicate those methods used in F3 and those red and green numbers indicate those methods used in ATMF.

Image	Metrics	Baseline	Standard	Demonet + GFPCA	GSA	HCM	SFIM	PCA	GFPCA	GLP	HPM	GS	PRACS	F3	ATMF	Best Score
Img1	PSNR	20.006	18.180	19.961	20.041	19.986	19.888	19.902	**20.873**	20.005	19.905	19.903	20.077	20.396	20.192	20.873
	Cielab	8.565	15.640	8.622	8.674	8.750	9.004	8.600	**7.482**	8.740	8.996	8.643	8.567	7.786	8.190	7.482
	SSIM	0.384	0.357	0.353	0.368	0.353	0.335	0.368	**0.432**	0.348	0.340	0.368	0.400	0.432	0.403	0.432
	HVS	14.494	13.248	14.562	14.523	14.493	14.514	14.328	**15.396**	14.523	14.514	14.404	14.525	14.902	14.651	15.396
	HVSm	14.607	13.338	14.647	14.632	14.612	14.636	14.431	**15.499**	14.641	14.635	14.511	14.628	14.988	14.747	15.499
Img2	PSNR	19.452	17.330	20.121	20.016	19.956	19.968	19.771	**23.036**	20.002	19.986	19.801	19.971	21.184	20.308	23.036
	Cielab	8.113	9.800	7.196	8.008	8.135	8.011	7.950	**4.715**	8.008	8.001	7.933	8.023	5.947	7.215	4.715
	SSIM	0.399	0.524	0.554	**0.604**	0.593	0.593	0.601	0.545	0.599	0.596	0.601	0.591	0.598	0.600	0.604
	HVS	14.772	11.877	15.691	15.089	15.053	15.084	14.893	**18.085**	15.100	15.083	14.837	15.054	16.439	15.458	18.085
	HVSm	15.082	12.013	15.882	15.307	15.278	15.317	15.095	**18.577**	15.328	15.317	15.047	15.281	16.695	15.682	18.577
Img3	PSNR	20.056	**20.564**	20.186	20.157	20.079	20.142	19.977	20.357	20.178	20.156	19.980	20.154	20.355	20.239	20.564
	Cielab	9.147	10.971	8.616	9.127	9.410	9.188	9.116	**7.899**	9.144	9.187	9.118	9.158	8.042	8.569	7.899
	SSIM	0.490	0.431	0.486	0.489	0.474	0.480	0.486	0.525	0.487	0.483	0.486	0.498	**0.527**	0.511	0.527
	HVS	14.957	15.233	15.275	15.027	15.011	15.058	14.857	15.239	15.059	15.061	14.860	15.018	**15.284**	15.120	15.284
	HVSm	15.117	**15.435**	15.386	15.167	15.157	15.204	14.995	15.369	15.203	15.207	14.999	15.159	15.399	15.247	15.435
Img4	PSNR	17.301	13.215	18.361	18.081	17.905	17.999	17.659	18.283	18.069	18.048	17.665	18.062	**18.644**	18.318	18.644
	Cielab	13.625	13.165	11.044	13.971	13.782	13.514	13.549	**8.185**	14.044	13.471	13.579	13.582	9.092	11.537	8.185
	SSIM	0.440	0.407	0.552	0.577	0.562	0.564	0.569	0.565	0.572	0.568	0.568	0.571	**0.602**	0.588	0.602
	HVS	12.619	8.090	**14.590**	13.121	13.050	13.172	12.666	13.515	13.183	13.177	12.621	13.010	14.133	13.472	14.590
	HVSm	13.240	8.306	**15.143**	13.661	13.614	13.749	13.163	14.050	13.751	13.754	13.119	13.557	14.666	14.006	15.143
Img5	PSNR	20.089	22.475	20.305	20.213	20.164	20.176	20.011	**27.076**	20.203	20.190	20.024	20.232	22.275	20.571	27.076
	Cielab	7.277	6.346	7.003	7.297	7.311	7.278	7.310	**3.337**	7.291	7.273	7.316	7.279	5.317	6.754	3.337
	SSIM	0.317	0.377	0.369	0.385	0.379	0.375	0.385	**0.440**	0.380	0.378	0.384	0.391	0.431	0.403	0.440
	HVS	15.802	17.356	16.304	15.942	15.909	15.947	15.704	**22.586**	15.950	15.943	15.697	15.925	18.081	16.311	22.586
	HVSm	16.006	17.653	16.442	16.117	16.088	16.131	15.867	**23.176**	16.133	16.129	15.866	16.099	18.283	16.479	23.176
Img6	PSNR	19.763	20.526	20.291	20.212	20.133	20.186	19.974	**25.261**	20.223	20.193	19.972	20.137	21.847	20.483	25.261
	Cielab	9.761	11.355	8.837	9.720	9.866	9.634	9.574	**4.999**	9.732	9.643	9.613	9.693	6.977	8.757	4.999
	SSIM	0.411	0.504	0.547	0.590	0.574	0.576	0.586	0.584	0.585	0.578	0.584	0.570	**0.603**	0.593	0.603
	HVS	15.392	16.551	16.064	15.619	15.622	15.685	15.426	**20.473**	15.676	15.691	15.427	15.565	17.387	15.960	20.473
	HVSm	15.692	16.911	16.253	15.845	15.847	15.913	15.657	**21.128**	15.905	15.920	15.652	15.810	17.673	16.198	21.128
Img7	PSNR	24.564	21.571	21.880	21.975	20.143	20.149	22.559	**25.615**	21.809	20.157	22.446	23.495	23.804	23.147	25.615
	Cielab	5.943	6.857	6.706	7.141	8.356	8.292	6.716	**4.024**	7.222	8.285	6.770	6.436	5.061	6.016	4.024
	SSIM	0.407	0.511	0.481	0.530	0.516	0.513	0.530	0.507	0.525	0.516	0.531	0.532	**0.544**	0.540	0.544
	HVS	20.236	17.426	17.841	17.554	15.768	15.782	18.128	**21.242**	17.404	15.779	17.993	19.014	19.558	18.729	21.242
	HVSm	20.730	17.619	18.006	17.752	15.897	15.917	18.355	**21.698**	17.605	15.915	18.216	19.300	19.811	18.967	21.698
Img8	PSNR	19.384	14.232	19.984	19.971	19.884	19.935	19.672	**21.107**	19.973	19.943	19.691	19.881	20.496	20.121	21.107
	Cielab	8.753	14.864	7.886	8.595	8.815	8.594	8.571	**6.139**	8.627	8.590	8.559	8.682	6.946	7.856	6.139
	SSIM	0.421	0.414	0.504	0.543	0.528	0.532	0.537	0.527	0.539	0.534	0.539	0.530	**0.555**	0.549	0.555
	HVS	14.576	8.817	15.441	14.928	14.911	14.973	14.652	**16.187**	14.974	14.973	14.630	14.867	15.682	15.171	16.187
	HVSm	14.874	8.900	15.623	15.143	15.130	15.200	14.857	**16.486**	15.200	15.202	14.840	15.097	15.899	15.388	16.486
Img9	PSNR	20.116	10.893	20.463	20.201	20.131	20.071	20.035	20.541	20.183	20.089	20.032	20.214	**20.560**	20.375	20.560
	Cielab	6.612	15.724	5.883	6.571	6.656	6.970	6.533	**5.224**	6.609	6.976	6.533	6.547	5.327	5.947	5.224
	SSIM	0.265	0.270	0.333	0.338	0.327	0.324	0.338	0.343	0.333	0.325	0.338	0.335	**0.359**	0.348	0.359
	HVS	15.468	6.264	**16.152**	15.560	15.535	15.574	15.401	15.875	15.575	15.575	15.406	15.538	16.001	15.726	16.152
	HVSm	15.686	6.294	**16.287**	15.753	15.737	15.775	15.589	16.016	15.774	15.776	15.593	15.735	16.138	15.894	16.287
Img10	PSNR	19.471	19.766	20.069	19.933	19.858	19.899	19.679	**20.694**	19.933	19.911	19.672	19.874	20.384	20.088	20.694
	Cielab	8.810	8.728	7.784	8.734	8.795	8.653	8.646	**6.476**	8.770	8.648	8.683	8.704	6.973	7.867	6.476
	SSIM	0.374	0.413	0.477	0.491	0.479	0.481	0.489	0.498	0.486	0.483	0.487	0.482	**0.518**	0.502	0.518
	HVS	15.573	15.812	16.434	15.836	15.843	15.905	15.575	**16.626**	15.884	15.905	15.592	15.787	16.488	16.055	16.626
	HVSm	15.926	16.202	16.640	16.097	16.102	16.170	15.840	**16.942**	16.152	16.172	15.851	16.067	16.736	16.316	16.942
Img11	PSNR	19.895	17.081	20.129	20.185	20.124	20.149	19.988	19.942	20.173	20.157	19.986	20.163	**20.200**	20.184	20.200
	Cielab	8.557	12.064	8.265	8.544	8.687	8.552	8.501	7.619	8.568	8.549	8.499	8.495	**7.577**	8.015	7.577
	SSIM	0.440	0.477	0.527	0.570	0.553	0.558	0.567	0.530	0.565	0.562	0.567	0.563	**0.576**	0.576	0.576
	HVS	14.937	11.423	**15.190**	15.053	15.042	15.073	14.886	14.849	15.071	15.076	14.882	15.038	15.137	15.084	15.190
	HVSm	15.123	11.508	**15.317**	15.201	15.194	15.226	15.030	14.998	15.224	15.229	15.026	15.190	15.267	15.222	15.317
Img12	PSNR	19.406	16.116	**20.155**	20.087	20.004	20.057	19.828	19.068	20.091	20.070	19.830	20.007	19.928	20.008	20.155
	Cielab	8.416	9.817	7.663	8.348	8.439	8.322	8.172	8.473	8.377	8.317	8.174	8.330	**7.472**	7.891	7.472
	SSIM	0.499	0.504	0.591	0.622	0.612	0.614	0.617	0.598	0.618	0.616	0.617	0.619	**0.632**	0.628	0.632
	HVS	15.138	11.211	**16.013**	15.381	15.372	15.425	15.131	14.657	15.426	15.428	15.132	15.347	15.512	15.427	16.013
	HVSm	15.490	11.347	**16.220**	15.640	15.634	15.695	15.383	14.863	15.694	15.699	15.385	15.619	15.724	15.672	16.220
Average	PSNR	19.959	17.662	20.159	20.089	19.864	19.885	19.921	**21.821**	20.070	19.900	19.917	20.189	20.839	20.336	21.821
	Cielab	8.632	11.278	7.959	8.728	8.917	8.834	8.603	**6.214**	8.761	8.828	8.618	8.625	6.877	7.885	6.214
	SSIM	0.404	0.432	0.481	0.509	0.496	0.495	0.506	0.508	0.503	0.498	0.506	0.507	**0.532**	0.520	0.532
	HVS	15.330	12.776	15.796	15.303	15.134	15.183	15.137	**17.061**	15.319	15.184	15.123	15.391	16.217	15.597	17.061
	HVSm	15.631	12.960	15.987	15.526	15.358	15.411	15.355	**17.400**	15.551	15.413	15.342	15.628	16.440	15.818	17.400

**Table A7:** Performance metrics of 14 algorithms at 20 dBs SNR (Poisson noise). Bold numbers indicate the best performing methods in each row. Red numbers indicate those methods used in F3 and those red and green numbers indicate those methods used in ATMF.

Image	Metrics	Baseline	Standard	Demonet + GFPCA	GSA	HCM	SFIM	PCA	GFPCA	GLP	HPM	GS	PRACS	F3	ATMF	Best Score
Img1	PSNR	19.835	19.873	20.240	19.459	19.739	19.588	19.369	**21.244**	19.444	19.734	19.283	19.412	20.325	19.555	21.244
	Cielab	9.075	8.939	7.816	8.571	8.301	8.715	8.504	**7.056**	8.634	8.593	8.630	8.575	7.664	8.311	7.056
	SSIM	0.339	0.345	0.425	0.459	0.458	0.463	0.454	0.435	**0.467**	0.464	0.454	0.444	0.457	0.459	0.467
	HVS	14.435	14.452	14.777	13.867	14.164	14.052	13.823	**15.777**	13.843	14.200	13.746	13.851	14.803	14.012	15.777
	HVSm	14.545	14.556	14.841	13.930	14.231	14.118	13.885	**15.862**	13.904	14.267	13.808	13.915	14.869	14.072	15.862
Img2	PSNR	19.541	19.942	**20.976**	18.936	18.833	19.497	18.958	20.783	19.328	19.441	18.900	19.002	20.440	19.340	20.976
	Cielab	8.903	8.298	**5.777**	8.106	8.200	7.710	7.945	5.883	7.851	7.754	7.964	8.029	6.210	7.352	5.777
	SSIM	0.477	**0.601**	0.429	0.550	0.545	0.557	0.541	0.418	0.557	0.555	0.540	0.518	0.485	0.527	0.601
	HVS	15.087	15.148	**16.727**	14.065	13.971	14.622	14.173	16.270	14.461	14.568	14.078	14.197	15.860	14.573	16.727
	HVSm	15.322	15.354	**16.984**	14.214	14.123	14.790	14.326	16.534	14.621	14.733	14.229	14.355	16.070	14.734	16.984
Img3	PSNR	19.889	19.965	**21.471**	19.893	20.157	20.180	19.822	21.395	20.125	20.132	19.904	19.987	21.027	20.188	21.471
	Cielab	9.724	9.532	7.055	8.705	8.615	8.510	8.678	**7.031**	8.559	8.560	8.615	8.654	7.299	8.142	7.031
	SSIM	0.459	0.472	0.558	0.562	0.559	0.568	0.554	0.555	0.570	0.568	0.555	0.549	**0.573**	0.565	0.573
	HVS	14.919	14.939	**16.531**	14.688	15.006	14.981	14.694	16.315	14.909	14.931	14.773	14.830	15.935	15.048	16.531
	HVSm	15.060	15.072	**16.653**	14.788	15.110	15.084	14.796	16.449	15.010	15.032	14.877	14.935	16.046	15.149	16.653
Img4	PSNR	17.405	17.944	**19.685**	17.759	17.804	17.748	17.485	18.901	17.758	17.839	17.522	17.855	19.012	17.926	19.685
	Cielab	15.784	15.404	**8.803**	13.954	13.631	13.641	13.570	9.380	14.108	13.579	13.562	13.431	9.722	12.096	8.803
	SSIM	0.473	0.558	0.595	0.601	0.592	0.594	0.593	0.586	0.598	0.597	0.593	0.591	**0.621**	0.610	0.621
	HVS	13.229	13.377	**15.841**	12.912	13.016	12.946	12.787	14.696	12.953	13.022	12.793	13.014	14.676	13.316	15.841
	HVSm	13.836	13.943	**16.458**	13.342	13.478	13.390	13.212	15.216	13.389	13.470	13.221	13.471	15.174	13.735	16.458
Img5	PSNR	19.942	20.022	**21.798**	20.122	19.746	19.916	20.298	21.657	19.946	19.400	20.152	20.318	21.149	20.403	21.798
	Cielab	7.929	7.701	**5.367**	6.837	7.058	6.970	6.632	5.422	6.974	7.352	6.724	6.675	5.751	6.406	5.367
	SSIM	0.326	0.369	0.332	0.370	0.365	0.371	0.362	0.330	**0.373**	0.369	0.362	0.356	0.355	0.364	0.373
	HVS	15.843	15.874	**17.760**	15.828	15.469	15.632	16.061	17.479	15.660	15.126	15.895	16.049	16.983	16.164	17.760
	HVSm	16.019	16.039	**17.918**	15.948	15.587	15.746	16.189	17.645	15.774	15.229	16.021	16.181	17.118	16.287	17.918
Img6	PSNR	19.772	20.076	**20.922**	20.175	20.028	20.148	19.994	20.344	20.120	19.981	19.964	19.984	20.526	20.086	20.922
	Cielab	10.727	10.270	**7.443**	8.869	9.037	8.835	9.009	7.760	9.001	8.991	9.075	8.975	7.703	8.444	7.443
	SSIM	0.473	**0.581**	0.403	0.521	0.511	0.525	0.503	0.395	0.526	0.522	0.500	0.478	0.457	0.493	0.581
	HVS	15.547	15.601	**16.711**	15.613	15.546	15.629	15.536	16.007	15.569	15.468	15.521	15.501	16.155	15.637	16.711
	HVSm	15.775	15.806	**16.947**	15.829	15.743	15.833	15.751	16.235	15.778	15.665	15.734	15.717	16.372	15.844	16.947
Img7	PSNR	19.847	19.985	29.058	26.350	21.086	20.872	25.651	**29.190**	25.921	20.208	26.357	22.369	28.456	27.001	29.190
	Cielab	9.161	8.743	**2.957**	4.699	7.064	7.227	4.843	3.011	4.855	7.718	4.629	6.294	3.249	4.158	2.957
	SSIM	0.419	0.511	0.462	0.560	0.526	0.531	0.548	0.450	**0.562**	0.521	0.551	0.513	0.513	0.549	0.562
	HVS	15.653	15.681	**26.259**	21.730	16.687	16.464	21.225	26.075	21.346	15.813	21.869	17.976	24.761	22.585	26.259
	HVSm	15.787	15.803	**27.349**	22.148	16.821	16.591	21.599	27.271	21.729	15.924	22.307	18.161	25.527	23.075	27.349
Img8	PSNR	19.438	19.846	19.835	19.319	19.355	19.193	19.503	**20.319**	19.396	19.115	19.339	19.293	19.931	19.448	20.319
	Cielab	9.564	9.027	7.249	8.423	8.448	8.521	8.140	**6.865**	8.421	8.591	8.262	8.450	7.215	7.869	6.865
	SSIM	0.445	0.531	0.416	0.522	0.519	0.524	0.509	0.417	**0.531**	0.523	0.508	0.490	0.472	0.502	0.531
	HVS	14.856	14.919	15.267	14.283	14.363	14.183	14.580	**15.657**	14.364	14.108	14.389	14.339	15.160	14.555	15.657
	HVSm	15.093	15.129	15.441	14.442	14.525	14.335	14.753	**15.861**	14.524	14.258	14.557	14.507	15.329	14.714	15.861
Img9	PSNR	20.041	20.098	**21.274**	19.210	19.487	19.078	18.979	20.657	19.442	19.143	19.031	19.085	20.486	19.452	21.274
	Cielab	7.247	6.991	**4.785**	6.386	6.230	6.770	6.417	5.097	6.323	6.751	6.390	6.432	5.143	5.925	4.785
	SSIM	0.286	**0.332**	0.303	0.322	0.318	0.317	0.320	0.293	0.325	0.317	0.320	0.305	0.308	0.316	0.332
	HVS	15.620	15.631	**16.878**	14.540	14.843	14.473	14.368	16.115	14.774	14.536	14.423	14.440	15.940	14.843	16.878
	HVSm	15.820	15.826	**16.995**	14.638	14.946	14.570	14.463	16.231	14.875	14.633	14.518	14.538	16.043	14.936	16.995
Img10	PSNR	19.519	19.827	**21.257**	19.511	19.212	19.411	19.314	20.395	19.603	19.455	19.254	19.213	20.483	19.599	21.257
	Cielab	9.597	9.095	**6.246**	8.418	8.615	8.436	8.360	6.780	8.439	8.408	8.444	8.548	6.768	7.811	6.246
	SSIM	0.411	**0.485**	0.411	0.466	0.457	0.469	0.457	0.397	0.472	0.469	0.453	0.437	0.441	0.455	0.485
	HVS	15.803	15.859	**17.678**	15.441	15.219	15.387	15.339	16.626	15.543	15.432	15.297	15.225	16.668	15.657	17.678
	HVSm	16.070	16.100	**17.936**	15.659	15.409	15.584	15.556	16.882	15.758	15.631	15.511	15.437	16.898	15.867	17.936
Img11	PSNR	19.815	20.030	19.751	19.717	19.591	19.827	19.644	**20.053**	19.844	19.804	19.537	19.634	19.934	19.632	20.053
	Cielab	9.263	8.863	7.810	8.190	8.302	8.109	8.126	**7.433**	8.134	8.133	8.211	8.216	7.549	7.931	7.433
	SSIM	0.472	**0.556**	0.375	0.501	0.493	0.510	0.488	0.373	0.513	0.510	0.486	0.464	0.437	0.472	0.556
	HVS	14.961	14.983	14.849	14.558	14.470	14.683	14.574	**15.101**	14.687	14.662	14.463	14.539	14.921	14.584	15.101
	HVSm	15.113	15.124	14.977	14.679	14.583	14.802	14.697	**15.245**	14.809	14.781	14.584	14.662	15.048	14.702	15.245
Img12	PSNR	19.572	**20.079**	19.241	19.740	19.654	19.760	19.599	19.093	19.697	19.685	19.557	19.566	19.422	19.369	20.079
	Cielab	9.242	8.757	7.950	7.951	7.988	7.914	7.796	7.927	8.036	7.977	7.831	8.012	**7.703**	7.828	7.703
	SSIM	0.529	0.610	0.530	0.614	0.611	0.617	0.598	0.522	**0.618**	0.616	0.598	0.589	0.570	0.589	0.618
	HVS	15.383	**15.429**	15.087	15.018	14.985	15.062	14.976	14.754	14.988	14.986	14.932	14.934	15.023	14.835	15.429
	HVSm	15.646	**15.673**	15.263	15.232	15.192	15.277	15.194	14.943	15.199	15.198	15.147	15.149	15.207	15.028	15.673
Average	PSNR	19.551	19.807	**21.292**	20.016	19.558	19.602	19.885	21.169	20.052	19.495	19.900	19.643	20.933	20.167	21.292
	Cielab	9.685	9.301	**6.605**	8.259	8.457	8.447	8.168	6.637	8.278	8.534	8.195	8.358	6.831	7.689	6.605
	SSIM	0.426	0.496	0.437	0.504	0.496	0.504	0.494	0.431	**0.509**	0.502	0.493	0.478	0.474	0.492	0.509
	HVS	15.111	15.158	**17.030**	15.212	14.812	14.843	15.178	16.739	15.258	14.738	15.181	14.908	16.407	15.484	17.030
	HVSm	15.340	15.369	**17.314**	15.404	14.979	15.010	15.368	17.031	15.447	14.902	15.376	15.086	16.642	15.679	17.314

**Table A8:** Performance metrics of 14 algorithms at 20 dBs SNR (Poisson noise). Bold numbers indicate the best performing methods in each row. Red numbers indicate those methods used in F3 and those red and green numbers indicate those methods used in ATMF.

Image	Metrics	Baseline	Standard	Demonet + GFPCA	GSA	HCM	SFIM	PCA	GFPCA	GLP	HPM	GS	PRACS	F3	ATMF	Best Score
Img1	PSNR	26.266	26.848	26.045	26.856	26.732	26.798	26.620	26.482	**26.932**	26.807	26.672	26.659	26.854	26.863	26.932
	Cielab	4.427	4.363	4.861	4.358	4.429	4.444	**4.301**	4.615	4.370	4.458	4.360	4.379	4.366	4.364	4.301
	SSIM	0.471	0.500	0.434	0.500	0.488	0.503	0.497	0.484	**0.504**	0.501	0.497	0.488	0.497	0.497	0.504
	HVS	21.159	21.329	20.686	21.389	21.355	21.432	21.045	21.124	21.441	**21.443**	21.234	21.329	21.387	21.401	21.443
	HVSm	21.587	21.685	20.956	21.731	21.705	21.777	21.357	21.417	21.785	**21.788**	21.567	21.696	21.729	21.741	21.788
Img2	PSNR	24.881	27.626	26.995	27.632	27.465	27.414	27.346	27.188	27.503	27.416	27.372	27.224	**27.649**	27.612	27.649
	Cielab	4.596	4.141	**3.222**	4.158	4.265	4.195	4.213	3.309	4.185	4.200	4.205	4.244	4.148	4.155	3.222
	SSIM	0.399	**0.586**	0.463	0.584	0.578	0.581	0.580	0.520	0.582	0.579	0.579	0.560	0.584	0.583	0.586
	HVS	21.028	22.441	**22.852**	22.537	22.394	22.484	22.629	22.752	22.596	22.481	22.259	22.370	22.518	22.538	22.852
	HVSm	22.408	23.591	**24.415**	23.667	23.557	23.690	23.743	24.196	23.796	23.695	23.329	23.542	23.653	23.682	24.415
Img3	PSNR	28.060	29.353	**30.667**	29.379	29.024	29.334	29.031	29.724	29.425	29.335	29.046	29.030	29.375	29.381	30.667
	Cielab	4.606	4.490	**3.731**	4.421	4.665	4.400	4.492	3.926	4.426	4.407	4.496	4.508	4.428	4.399	3.731
	SSIM	0.563	0.609	0.580	0.609	0.598	0.608	0.604	**0.615**	0.609	0.607	0.604	0.596	0.607	0.607	0.615
	HVS	23.811	24.302	**26.429**	24.380	24.317	24.355	24.110	25.147	24.334	24.354	24.092	24.268	24.379	24.403	26.429
	HVSm	24.911	25.215	**27.665**	25.287	25.255	25.314	24.955	26.012	25.291	25.318	24.943	25.213	25.286	25.315	27.665
Img4	PSNR	18.786	20.395	20.640	20.450	20.215	20.343	19.984	**20.845**	20.398	20.377	19.987	20.208	20.452	20.434	20.845
	Cielab	12.202	12.236	**6.391**	11.990	11.882	11.642	11.773	6.786	12.140	11.624	11.801	11.809	11.981	11.750	6.391
	SSIM	0.456	0.636	0.614	0.636	0.619	0.625	0.625	0.631	0.629	0.626	0.624	0.613	**0.636**	0.634	0.636
	HVS	14.151	14.843	**16.086**	14.948	14.885	15.024	14.567	15.990	15.034	15.036	14.488	14.765	14.948	14.963	16.086
	HVSm	15.014	15.558	**16.904**	15.661	15.626	15.786	15.236	16.813	15.789	15.803	15.154	15.506	15.661	15.682	16.904
Img5	PSNR	27.318	28.808	**29.054**	28.825	28.634	28.749	28.383	28.744	28.818	28.748	28.448	28.546	28.829	28.816	29.054
	Cielab	3.399	3.270	**2.950**	3.204	3.243	3.216	3.330	3.046	3.220	3.218	3.305	3.243	3.206	3.201	2.950
	SSIM	0.325	0.389	0.346	**0.389**	0.385	0.389	0.385	0.375	0.387	0.386	0.384	0.378	0.388	0.388	0.389
	HVS	23.466	24.370	**24.902**	24.446	24.363	24.536	24.014	24.512	24.568	24.538	23.966	24.273	24.442	24.488	24.902
	HVSm	24.548	25.213	**25.890**	25.278	25.227	25.426	24.756	25.353	25.447	25.433	24.734	25.154	25.275	25.325	25.890
Img6	PSNR	25.857	28.142	**28.335**	28.213	27.878	28.024	27.841	28.194	28.141	27.992	27.851	27.708	28.214	28.179	28.335
	Cielab	5.375	5.111	4.159	4.964	5.105	4.926	5.204	**4.111**	5.022	4.952	5.223	5.073	4.974	4.939	4.111
	SSIM	0.418	**0.568**	0.477	0.568	0.559	0.567	0.561	0.531	0.567	0.564	0.559	0.537	0.567	0.567	0.568
	HVS	22.006	23.068	**23.951**	23.067	23.069	23.157	22.646	23.579	23.199	23.177	22.799	22.793	23.091	23.145	23.951
	HVSm	23.434	24.276	**25.353**	24.295	24.302	24.438	23.809	24.871	24.484	24.466	23.971	24.081	24.313	24.370	25.353
Img7	PSNR	26.971	28.543	27.003	28.579	28.490	28.436	**28.706**	28.500	28.475	28.419	28.699	28.403	28.578	28.560	28.706
	Cielab	4.068	3.765	3.589	3.772	3.823	3.802	3.755	**3.268**	3.783	3.807	3.747	3.827	3.774	3.777	3.268
	SSIM	0.437	**0.590**	0.473	0.588	0.584	0.587	0.585	0.538	0.587	0.585	0.585	0.573	0.587	0.586	0.590
	HVS	23.317	24.178	23.444	24.239	24.212	24.170	24.462	**24.712**	24.206	24.159	24.348	24.160	24.237	24.246	24.712
	HVSm	24.260	24.908	24.172	24.977	24.967	24.951	25.234	**25.621**	24.980	24.942	25.112	24.926	24.976	24.990	25.621
Img8	PSNR	25.298	28.544	27.792	**28.723**	28.383	28.325	28.265	27.953	28.416	28.276	28.314	28.087	28.723	28.677	28.723
	Cielab	4.431	4.120	**3.250**	4.015	4.161	4.034	4.114	3.262	4.053	4.044	4.090	4.157	4.018	4.009	3.250
	SSIM	0.453	0.571	0.477	**0.571**	0.563	0.570	0.561	0.537	0.571	0.568	0.561	0.548	0.570	0.570	0.571
	HVS	21.405	22.734	23.977	23.371	23.370	23.361	23.214	**24.116**	23.332	23.369	22.960	22.951	23.353	23.416	24.116
	HVSm	22.819	23.953	25.568	24.639	24.691	24.740	24.414	**25.658**	24.716	24.770	24.134	24.239	24.620	24.698	25.658
Img9	PSNR	26.606	27.968	27.180	28.051	27.819	27.839	27.779	**28.388**	28.019	27.745	27.771	27.812	28.047	28.048	28.388
	Cielab	3.649	3.564	3.242	3.469	3.524	3.632	3.521	**2.993**	3.514	3.738	3.523	3.497	3.477	3.465	2.993
	SSIM	0.264	0.323	**0.345**	0.323	0.317	0.318	0.322	0.325	0.316	0.311	0.322	0.313	0.327	0.326	0.345
	HVS	22.272	22.829	22.590	23.017	22.987	23.133	22.751	**23.768**	23.146	23.146	22.774	22.846	23.015	23.066	23.768
	HVSm	23.228	23.573	23.141	23.746	23.728	23.893	23.446	**24.480**	23.903	23.909	23.467	23.621	23.745	23.796	24.480
Img10	PSNR	24.774	26.876	25.963	26.915	26.681	26.815	26.514	**27.096**	26.883	26.804	26.512	26.459	26.919	26.908	27.096
	Cielab	5.106	4.821	3.933	4.762	4.848	4.730	4.864	**3.731**	4.811	4.737	4.843	4.833	4.765	4.732	3.731
	SSIM	0.375	0.482	0.431	0.482	0.474	0.483	0.478	0.465	**0.483**	0.481	0.475	0.461	0.482	0.482	0.483
	HVS	21.234	22.249	22.291	22.283	22.350	22.427	21.779	**23.305**	22.400	22.421	21.958	22.054	22.305	22.375	23.305
	HVSm	22.565	23.299	23.324	23.357	23.415	23.538	22.796	**24.565**	23.522	23.542	22.981	23.201	23.370	23.439	24.565
Img11	PSNR	26.150	27.606	26.454	**27.650**	27.499	27.611	27.389	27.309	27.647	27.596	27.386	27.321	27.648	27.646	27.650
	Cielab	4.713	4.534	4.568	4.505	4.600	4.510	4.538	**4.267**	4.518	4.514	4.536	4.547	4.511	4.502	4.267
	SSIM	0.421	0.524	0.416	0.524	0.517	0.529	0.519	0.496	**0.530**	0.529	0.518	0.500	0.523	0.524	0.530
	HVS	22.398	22.906	21.891	23.125	23.164	23.220	22.867	23.069	23.225	**23.238**	22.859	22.963	23.124	23.164	23.238
	HVSm	23.440	23.781	22.668	23.984	24.018	24.097	23.687	23.963	24.108	**24.118**	23.678	23.866	23.983	24.020	24.118
Img12	PSNR	22.103	23.523	**24.317**	23.555	23.418	23.507	23.243	23.423	23.558	23.513	23.245	23.317	23.554	23.530	24.317
	Cielab	5.753	5.514	**4.447**	5.488	5.541	5.468	5.435	4.788	5.506	5.470	5.435	5.519	5.495	5.479	4.447
	SSIM	0.508	**0.647**	0.559	0.647	0.641	0.646	0.637	0.616	0.646	0.644	0.637	0.629	0.646	0.645	0.647
	HVS	18.482	18.928	**20.675**	19.040	19.045	19.115	18.743	19.380	19.126	19.125	18.742	18.951	19.040	19.047	20.675
	HVSm	19.240	19.512	**21.479**	19.603	19.605	19.697	19.285	19.970	19.707	19.709	19.284	19.546	19.603	19.609	21.479
Average	PSNR	25.256	27.019	26.704	27.069	26.853	26.933	26.758	26.987	27.018	26.919	26.775	26.731	**27.070**	27.054	27.070
	Cielab	5.194	4.994	4.029	4.926	5.007	4.917	4.961	**4.008**	4.962	4.931	4.964	4.970	4.929	4.898	4.008
	SSIM	0.424	**0.535**	0.468	0.535	0.527	0.534	0.529	0.511	0.534	0.532	0.529	0.516	0.534	0.534	0.535
	HVS	21.227	22.015	22.481	22.153	22.126	22.201	21.902	**22.621**	22.217	22.207	21.873	21.977	22.153	22.188	22.621
	HVSm	22.288	22.880	23.461	23.019	23.008	23.112	22.726	**23.577**	23.127	23.124	22.696	22.883	23.018	23.055	23.577
